# Pitfall Flower Development and Organ Identity of *Ceropegia sandersonii* (Apocynaceae-Asclepiadoideae)

**DOI:** 10.3390/plants9121767

**Published:** 2020-12-14

**Authors:** Annemarie Heiduk, Dewi Pramanik, Marlies Spaans, Loes Gast, Nemi Dorst, Bertie Joan van Heuven, Barbara Gravendeel

**Affiliations:** 1 School of Life Sciences, University of KwaZulu-Natal, Private Bag X01, Scottsville, Pietermaritzburg 3209, South Africa; annemarie.heiduk@gmx.net; 2 Naturalis Biodiversity Center, Darwinweg 2, 2333 CR Leiden, The Netherlands; dewi.pramanik@naturalis.nl (D.P.); bertiejoan.vanheuven@naturalis.nl (B.J.v.H.); 3 Institute of Biology Leiden, Leiden University, Sylviusweg 72, 2333 BE Leiden, The Netherlands; 4 Indonesian Ornamental Crops Research Institute (IOCRI), Jl. Raya Ciherang, Pacet-Cianjur 43253, Indonesia; 5 Faculty of Science and Technology, University of Applied Sciences Leiden, Zernikedreef 11, 2333 CK Leiden, The Netherlands; marlies.spaans1@gmail.com (M.S.); gastloes@gmail.com (L.G.); nemi.dorst@wur.nl (N.D.); 6 Institute of Water and Wetland Research, Radboud University, Heyendaalseweg 135, 6500 GL Nijmegen, The Netherlands

**Keywords:** *Ceropegia*, MADS-box genes, micro-CT scanning, RT-PCR, vascularization, SEM, transcriptomics

## Abstract

Deceptive *Ceropegia* pitfall flowers are an outstanding example of synorganized morphological complexity. Floral organs functionally synergise to trap fly-pollinators inside the fused corolla. Successful pollination requires precise positioning of flies headfirst into cavities at the gynostegium. These cavities are formed by the corona, a specialized organ of corolline and/or staminal origin. The interplay of floral organs to achieve pollination is well studied but their evolutionary origin is still unclear. We aimed to obtain more insight in the homology of the corona and therefore investigated floral anatomy, ontogeny, vascularization, and differential MADS-box gene expression in *Ceropegia sandersonii* using X-ray microtomography, Light and Scanning Electronic Microscopy, and RT-PCR. During 10 defined developmental phases, the corona appears in phase 7 at the base of the stamens and was not found to be vascularized. A floral reference transcriptome was generated and 14 MADS-box gene homologs, representing all major MADS-box gene classes, were identified. B- and C-class gene expression was found in mature coronas. Our results indicate staminal origin of the corona, and we propose a first ABCDE-model for floral organ identity in *Ceropegia* to lay the foundation for a better understanding of the molecular background of pitfall flower evolution in Apocynaceae.

## 1. Introduction

Flowering plants are the dominant plants on earth with a breathtaking diversity in floral forms and functions. Variability in the number, form, function, and arrangement of floral organs in different whorls allowed for immense diversification during the past ~130 million years. In higher angiosperms, numerical reduction of floral whorls led to fusion and/or mechanical linkage of separate floral organs within and between whorls ([1,2,3], see also [4]). This synorganization resulted in further floral complexity [4,5].

Among the 10 largest plant families, the milkweeds (Apocynaceae) are remarkable with regard to diversity in growth form, flower morphology, and pollination strategies [6]. Especially species in the subfamily Asclepiadoideae show extreme floral complexity, high levels of floral synorganization, and huge diversity in pollinator usage [6,7]. Asclepiadoideae flowers are outstanding among the dicotyledonous angiosperms [8], just as flowers of Orchidaceae are among the monocotyledons. This is why they are sometimes referred to as “dicotyledonous orchids” ([5], see also [9]). In Asclepiadoideae, the flowers are pentamerous with fixed numbers of floral organs, i.e., two carpels, and five synandrous stamens, petals (with various degrees of basal sympetaly [10]), and sepals [5]. The floral organs are tangentially (within the same whorl) and radially (between two whorls) synorganized by either congenital fusion, i.e., congenital connation of organ meristems, which then arise confluent, or postgenital fusion, i.e., union of already differentiated organs (for details see [8,11,12]). Male (androecium) and female (gynoecium) reproductive organs are synorganized via postgenital fusion into a highly specialized, radially symmetric and compound structure called the gynostegium [5]. The gynostegium is embraced by the corona, a unique floral organ between corolla and androecium. In Asclepiadoideae, this organ is typically either a result of synorganization between the corolla and the stamens, or entirely develops from stamen derived tissue [10,13]; the corona plays an important role in the pollination process. The ultimate floral complexity is highlighted by pollen transfer via pollinia, i.e., compact pollen packages. Two such packages are interconnected via translator arms (secreted by the style-head; see [5]) to form a pollinarium, which is attached to the pollinator’s body via a mechanical clip, i.e., the corpusculum (see [5]). The floral complexity in Apocynaceae stimulated comparative studies of flower structure and ontogenetic development [7,10,13,14,15,16,17]. Within Asclepiadoideae, a high point of functional synorganization is represented by the sophisticated pitfall flowers in the genus *Ceropegia*. In addition to the complexity of the synorganized reproductive organs within Asclepiadoideae, the flowers of *Ceropegia* have con- and postgenitally fused petals resulting in a tubular, funnel-shaped corolla (see [7]). The corolla lobe tips form a cage-like structure with five openings through which insects can enter. They further comprise osmophoric tissue for olfactory attraction of pollinating flies, as well as surfaces promoting the trapping of attracted flies (see below). In many species the corolla lobe tips are equipped with motile trichomes, likely to attract flies (see [18]). The cylindrical flower tube has smooth inner surfaces and downward-pointing hairs to block the exit, and to temporarily detain flies inside the basal corolla inflation which encloses the gynostegium. Successful pollination requires insertion of a pollinium (from a pollinarium clipped to a fly in a donor flower), into one of the five lateral guide rails on the gynostegium of a receptive flower. For uptake of a pollinarium and insertion of a pollinium, the pollinating fly needs to be in contact with the gynostegium in a precise way comparable to a “key-lock pair”. The corona structure around the gynostegium positions a fly head over heels, and proboscis extension into the coronal nectar cavities finally leads to pollinaria uptake and/or pollinia insertion. Only a fly species with proportionate body size and mouthparts will fit, and thus the corona structure ultimately acts as a species-specific pollinator filter to achieve reproductive isolation. 

The functional morphology of these elaborate pollinator trap flowers is a result of many adaptations towards optimization of floral organs into specific shapes and at particular positions [5]. *Ceropegia* flowers aroused the interest of naturalists more than a century ago [19,20], but most detailed descriptions of floral structure, functional floral parts and tissues, and their complex interaction with flies to achieve pollination were published by Stefan Vogel [18,21,22]. Only recently, studies on chemical ecology of *Ceropegia* species [23,24,25,26,27] elucidated fascinating chemical mimicry strategies such as kleptomyiophily, i.e., mimicry of injured or dead insects as specific food items of kleptoparasitic flies. Apparently, combined floral chemistry and morphology are the key to pollinator attraction and successful pollination of *Ceropegia*. The chemistry received quite some attention from researchers [23,24,25,26,27] but ontogeny and organ development of *Ceropegia* pitfall flowers are still not fully understood. The molecular processes controlling floral development have not yet been studied, and genes that are involved in the formation of specific floral organs are unknown. The species-specific corona is an important character by which different species can be distinguished [13]. Despite its relevance for taxonomy, the ontogeny of the corona was only studied in a few *Ceropegia* species and exclusively using anatomical methods [8,14].

The objectives of the present study were to investigate the origin of the corona in *Ceropegia sandersonii* Decne. ex Hook. f. pitfall flowers combining anatomical and molecular methods. We specifically asked whether the corona is a result of synorganization between the corolla and the stamens, or entirely made up from stamen derived tissue. We furthermore aimed to illustrate and classify floral organ formation from primordia to mature flowers to provide a framework for further comparative developmental studies of *Ceropegia* pitfall flowers and other closely related taxa.

## 2. Results

### 2.1. Anatomy of Mature Pitfall Flowers

**Figure 1:**
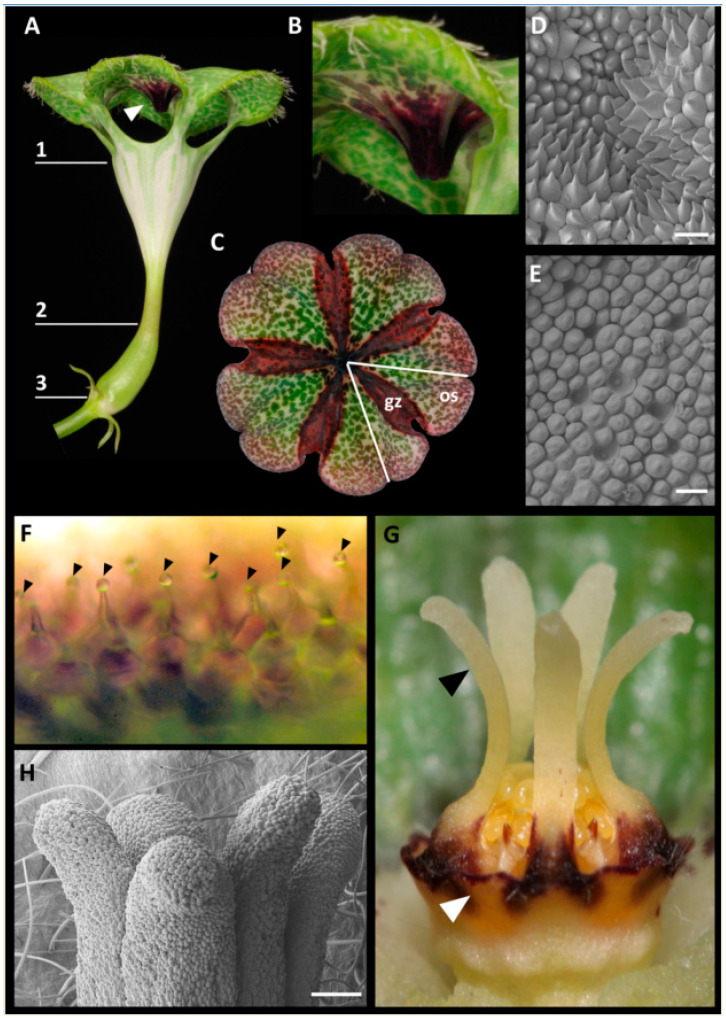
Morphology of *Ceropegia sandersonii* trap flowers. (**A**) The parachute-like tubular corolla is formed by fusion of five petals and has three functional parts, i.e., (**1**) the umbrella-like corolla tip, (**2**) the funnel-shaped corolla tube, and (**3**) the inflated base enclosing the synorganized reproductive organs, i.e., gynostegium. The white arrowhead indicates the uvula, a purple protrusion on the underside of the umbrella-like corolla tip; (**B**) close up of the uvula; (**C**) umbrella-like corolla tip stained with neutral red. Each of the five corolla lobes (one lobe separated by white lines) comprises osmophoric tissue (os) and gliding zones (gz; stained with neutral red); (**D**) SEM image of the epidermal surface of the uvula; (**E**) SEM image of the osmophoric cells on the corolla lobes; (**F**) LM image of the conical acuminate to caudate epidermis cells of the gliding zones on the corolla lobes with liquid droplets at their tips (magnification: 160×); (**G**) gynostegium formed by fusion of male and female reproductive organs. It is surrounded by the corona with slender and apically spreading staminal corona lobes (black arrowhead) and interstaminal corona lobes forming nectar cups (white arrowhead); (**H**) SEM image of the globular epidermis cells of staminal corona lobes of a mature flower. Abbreviations: os—osmophoric zone, gz—gliding zone. Scale bars: 30 µm in (**D**,**E**), 200 µm in **H**. Photographs by AH and David Styles.

In *Ceropegia sandersonii* trap flowers (Figure 1A), the corolla is fused into a funnel-shaped tube roofed by an umbrella-shaped structure formed by unusual postgenital fusion and disproportional growth of the five corolla lobes (see [18,21]). The uvula, a dark-purple protuberance on the underside of the umbrella in its center right above the throat of the floral tube (Figure 1A,B) is a peculiar result of this disproportionate growth (see [21]). In the umbrella, each of the five corolla lobes comprises two different types of zones, osmophoric zones (os; Figure 1C) from which scent is released (see [8,18]), and so-called gliding zones (gz; Figure 1C; see [18,21]), which include the uvula. The surfaces of both these zones have distinct epidermal cells: The gliding zones have acuminate to caudate cells (Figure 1D) which stained positively with neutral red (Figure 1C), while the osmophoric zones have a papillate epidermis (Figure 1E) not stainable with neutral red (Figure 1C). Flower-visiting flies that walk onto the uvula or the gliding zones were observed to slip off and fall into the funnel-shaped corolla throat and down through the corolla tube to become trapped inside the basal inflation (own obs., see also [18,21]). When viewed under a light microscope, the gliding zones of newly opened flowers carried little liquid droplets at their tips (Figure 1F); however, no secretory residues were seen when studying gliding zones using SEM. The staminal corona lobes of the gynostegium (Figure 1G, black arrowhead) have globular epidermis cells (Figure 1H).

### 2.2. Early Floral Bud and Gynostegium Development

**Figure 2:**
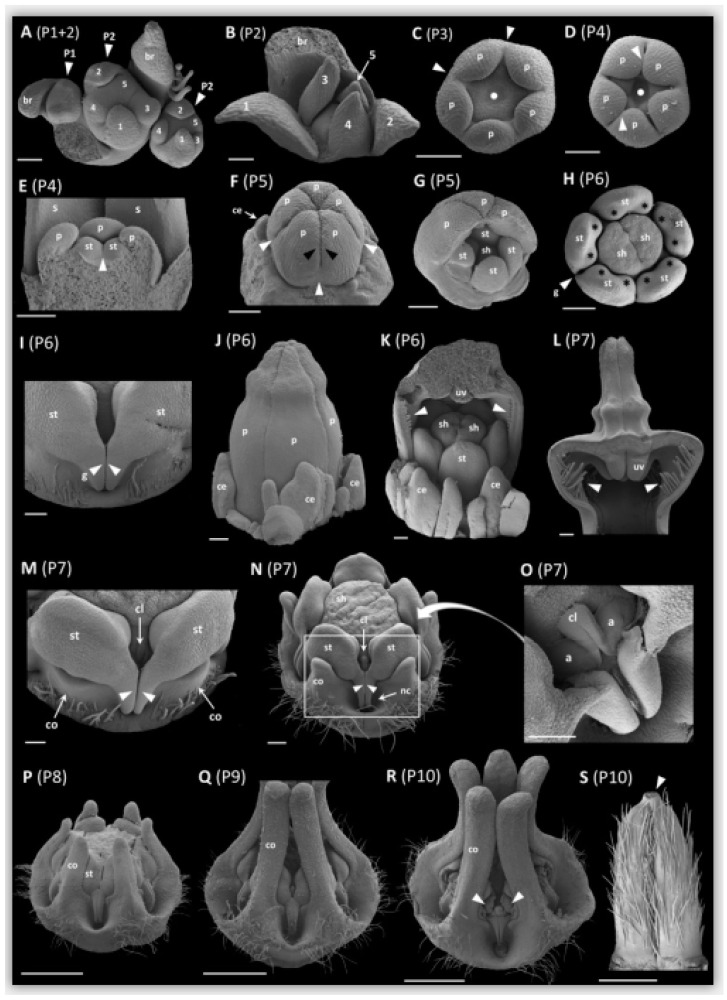
Ten different developmental phases (**P**) of *Ceropegia sandersonii* flowers from initiation to synorganization of floral organs. (**A**) shoot apex with three developing flowers (indicated by arrowheads), one thereof in developmental phase P1, and two in phase P2 with sepals initiating in a sequence from first (1) to last (5); (**B**) sequentially developing sepals in late phase P2 with numbers 1–5 indicating the sequence of development; (**C**) bud in P3 (sepals removed) with early congenital fusion (white arrowheads) of petal bases and a slight central depression (filled circle); (**D**) bud in P4 (sepals removed) with distinct central depression (filled circle); petals show first signs of postgenital fusion at their tips (white arrowheads); (**E**) cross section of bud in phase P4 revealing stamen initiation; sepals visible in the background; (**F**) bud in phase P5 with corolla fully enclosing the developing reproductive organs. Petals are congenitally fused at bases (white arrowheads) and entirely postgenitally fused at upper parts with valvate margins (one thereof indicated by black arrowheads). Colleters begin to develop; (**G**) bud in P5 with two petals removed revealing the early stamens and the developing halves of the style-head in the center; (**H**) top view of developing gynostegium in P6 showing the clearly dimerous style-head with a median groove. Each stamen shows two slight bulges (black asterisks) as a first indication of pollinia development. The anther wings of two neighboring stamens start forming a guide rail (white arrowhead); (**I**) close-up side view of guide rails indicated by white arrowhead in (**H**); (**J**) bud in P6; the corolla is cylindrical due to length growth of postgenitally fused apical parts of petals; (**K**) bud in P6 with two petals removed revealing early stamen, the dimerous style-head, and the early stage motile trichomes (white arrowheads) on edges of petal tips. The uvula starts to develop; colleters are prominent; (**L**) cross section through upper part of corolla in P7. The postgenitally fused corolla lobe tips start expanding into the umbrella-like structure typical for *C. sandersonii*; (**M**) close-up oblique view of two neighboring stamens in early P7 with corona lobes initiating at the base of each stamen. Above each guide rail, a corpusculum is visible (white frame in (**N**) indicates the position of the magnified section shown); (**N**) gynostegium in late P7 with the corona lobes growing up along the dorsal sides of stamens. Interstaminal corona parts form the coronal nectar cavities underneath each guide rail (arrowheads); (**O**) close-up oblique view on a corpusculum in P7. Curved arrow back to (**N**) indicates the position of the magnified part. The two translator arms are almost fully secreted; (**P**) gynostegium in late P8 with stamens and corona lobes being postgenitally fused. Corona lobe tips outgrow the stamens. Nectar cavities are deep cups; (**Q**) gynostegium in P9 with corona lobes at least half as long as the height of the gynostegium; (**R**) side view of mature gynostegium in P10. Pollinia (white arrowheads) are released from the thecae, and two pollinia are interconnected via translator arms and the corpusculum to form a pollinarium, one sitting above each guide rail; (**S**) mature carpels with postgenitally fused apices (white arrowhead). Abbreviations: a—translator arm, br—bract, ce—colleter, cl—corpusculum, co—corona, g—guide rail, nc—nectar cavity, p—petal, s—sepal, sh—style-head, st—stamen, uv—uvula. Scale bars: 100 µm in A–G, J, I, M, O; 200 µm in H, K, N, L; 1 mm in P–S. Images by AH.

**Figure 3:**
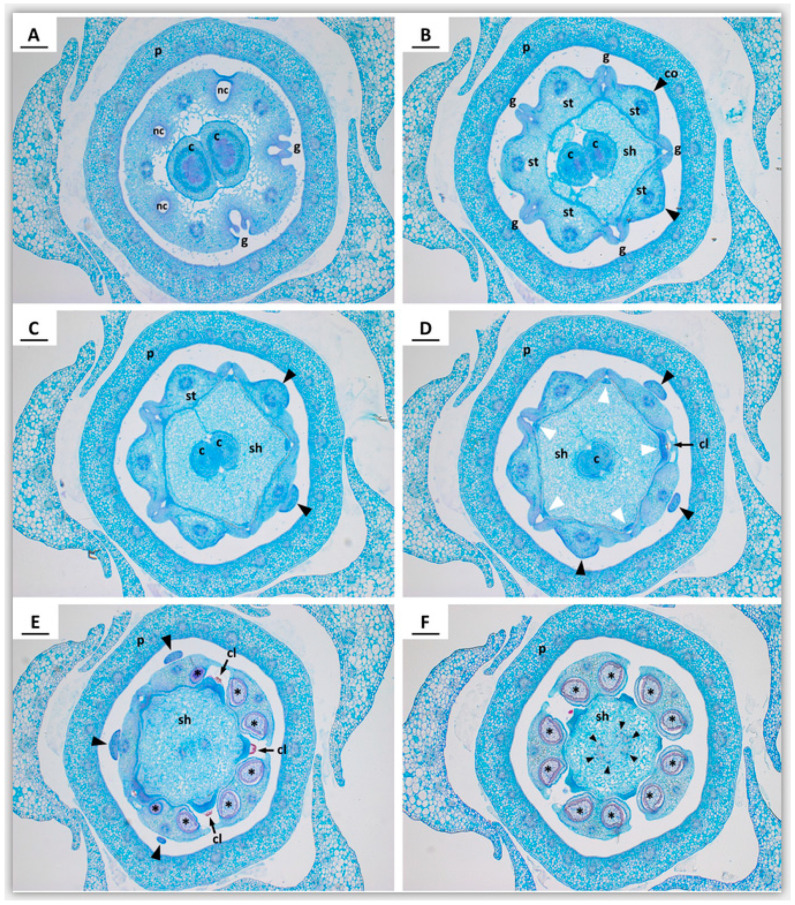
Transverse microtome sections through the gynostegium in a *Ceropegia sandersonii* flower bud in developmental phase P7. (**A**) Section showing three out of the five nectar cavities formed by interstaminal corona tissue; (**B**) section at level of the guide rails formed by anther flanks of two neighbouring stamens. Staminal corona tissue is formed on the dorsal side of stamens (black arrowheads); (**C**) section at level where staminal corona lobes (black arrowheads) separate from the stamen; (**D**) at level of the corpuscula, which are secreted by the translator glands (white arrowheads) of the style-head. The carpel apices are postgenitally fused; (**E**) at level of the pollinia (black asterisks; see also **F**) produced by the anthers; (**F**) at level of branched vascular bundles in the style-head (black arrowheads). The microtome sections were stained using Etzold’s solution. Abbreviations: c—carpel, cl—corpusculum, co—corona, g—guide rail, p—petals (fused), sh—style-head, st—stamen. Scale bars: 200 µm. Images by B.J.v.H.

We defined 10 phases (P1 to P10) during ontogeny of *Ceropegia sandersonii* trap flowers with distinct changes in organ development. Phase P1 (Figure 2A) is the emergence of a floral primordium adaxially positioned to a subtending floral bract. Phase P2 (Figure 2A) is defined by presence of the five young sepals in sequential order from first to last (1–5; Figure 2A). During subsequent sequential sepal development (Figure 2A,B), the second sepal develops approximately opposite of the first, the third and fourth develop on both sides of the first sepal, and the fifth sepal develops between the second and the third (Figure 2A,B). In the third phase P3, the corolla starts to develop with simultaneous initiation of the five petal primordia in penta-symmetrical order and alternating with the sepals. In a later stage of P3, the petal bases show early congenital fusion (white arrowheads; Figure 2C). The floral apex in the center of the emerging petals shows a depression (filled white circles; Figure 2C,D). In phase P4 (Figure 2D,E), the central depression becomes more distinct and the five stamen (st; Figure 2E) emerge simultaneously in penta-symmetrical order. In later P4, the stamens show early congenital fusion (white arrowhead; Figure 2E). The petals start to become valvate and show the first signs of postgenital fusion towards their upper parts leaving pouches at the petal sinuses (white arrowheads; Figure 2D). In phase P5 (Figure 2F,G), the petals fully enclose the developing gynostegium with congenitally fused petal bases (white arrowheads; Figure 2F) and postgenitally fused valvate upper parts (black arrowheads; Figure 2F). In this developmental phase, the style-head (sh; Figure 2G) develops with two separated halves above the two carpels. The colleters (ce; Figure 2F), i.e., glandular outgrowths of the sepals (see [28]), start to develop in unsymmetrical order. In phase P6 (Figure 2H–K), the corolla is cylindrical and mainly formed by the postgenitally fused petal apices (Figure 2J). Motile hairs, which in mature flowers dangle from the edges of the umbrella-shaped flower tip, are already distinct (white arrowheads; Figure 2K). The uvula (uv; Figure 2K; see also Figure 1B), a cone-like structure formed by postgenital fusion and inward-folding of the corolla lobe tips (see [21]), develops. In this phase, the style-head (sh; Figure 2H) is already well developed and clearly separated from the stamens (st; Figure 2H); it is dimerous with a median groove formed by the two carpels (see [5]). Furthermore, in P6, the anther wings of two neighboring stamens start forming guide rails (g; white arrowheads; Figure 2H,I), and each stamen shows two bulges indicating the development of a pollinium in each pollen sac (black asterisks; Figure 2H); colleters are prominent (ce; Figure 2J,K). In phase P7 (Figure 2L–O and Figure 3A–F), the upper part of the corolla starts expanding to form the umbrella-like flower tip (Figure 2L). The motile hairs are well developed (white arrowheads; Figure 2L) and the uvula is maturing (uv; Figure 2L). The corona (co; Figure 2M) appears with one lobe each at the base of a stamen congenitally fused with the latter. Above each guide rail (white arrowheads; Figure 2M), a corpusculum (cl; Figure 2M; see also Figure 3D,E) is secreted by the style-head. In later P7, the five staminal corona lobes grow one each along the dorsal side of a stamen (co; Figure 2N; see also Figure 3B–D; black arrowheads), and the interstaminal corona parts form nectar cavities (nc; Figure 2N; see also Figure 3A) one underneath each guide rail (white arrowheads; Figure 2N). The translator arms (a; Figure 2O), which connect pollinia of two neighboring anthers to the corpusculum (cl; Figure 2O), are secreted by the style-head; the latter is postgenitally fused with the anthers in this phase (Figure 2N; see also Figure 3B–E). The pollinia (black asterisks; Figure 3E,F) are well developed inside the thecae. The median groove on the style-head disappears and it molds from di- to penta-symmetry as the two carpel tips underneath become postgenitally fused (c; Figure 3D). In phase P8 (Figure 2P), the corona is fully postgenitally fused with the anthers. The corona lobes continue to elongate apically and outgrow the stamens with the result that the corona now embraces the remainder of the gynostegium. The nectar cavities become deeper. In phase P9 (Figure 2Q), the pollinia from neighboring anthers become connected via the corpusculum to form a pollinarium; the corona lobes are at least as long as the height of the gynostegium; their upper parts touch each other laterally, however, they remain unfused and their very tips are slightly spreading. In the final phase P10 (Figure 2R), the thecae mature and display the pollinia (white arrowheads; Figure 2R). The corpusculum and its two translator arms have hardened and the pollinaria are fully developed. The two mature carpels (Figure 2S) are postgenitally fused at their tips (white arrowhead; Figure 2S); they are densely covered with single-celled erect trichomes of up to 0.5 mm in length.

### 2.3. Vascularization in Mature Flowers

**Figure 4:**
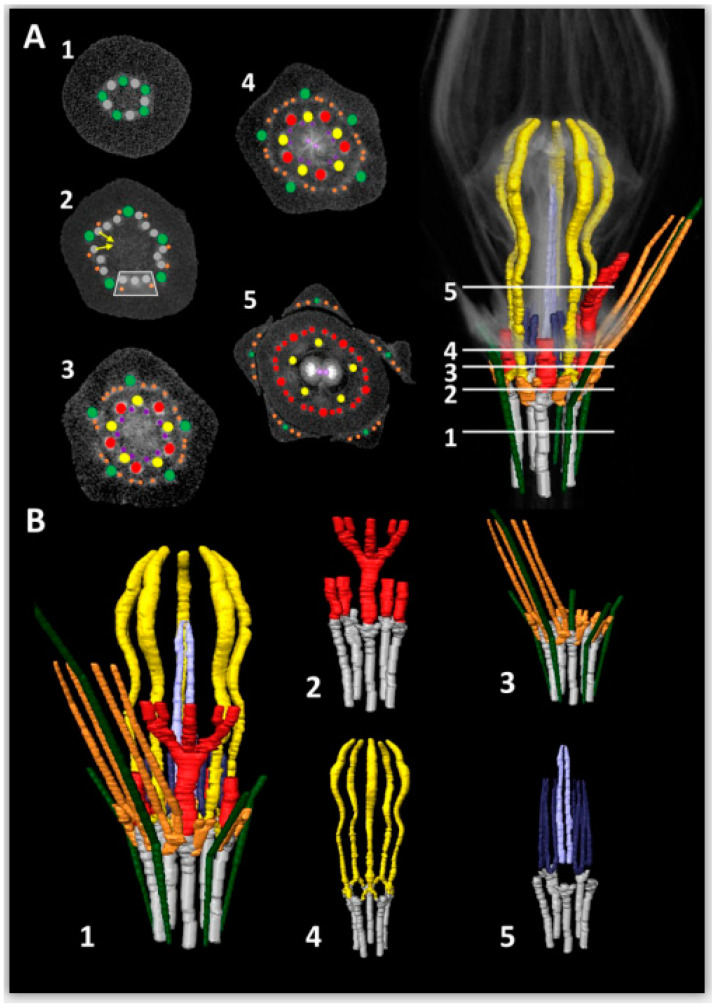
Reconstruction of vascular bundles in mature *Ceropegia sandersonii* flowers based on 3D X-ray micro-CT scanning. (**A**) 3D scan of hypanthium showing cross sections (**1**–**5**) positioned where major changes in vascularization occur. Arrows in (**2**) indicate the merging event resulting in stamen bundles; the white rectangle indicates one of five bundle triplets alternating with the main sepal bundles; (**B**) full 3D model of vascular bundles (**1**), and partial 3D models of petal bundles (**2**), sepal bundles (**3**), stamen bundles (**4**), and carpel bundles. Colors refer to tissue specific vascular bundles as follows: Grey—pedicel, green—sepals (main bundles), orange—sepals (secondary bundles), red—petals, yellow—stamens, dark and light purple—carpels. See also: 3D Movie (Movie S1).

In the micro-CT scanning and Light Microscopy (LM) analyses of vascularization in *Ceropegia sandersonii* flowers, we discerned a vascular cylinder of 10 (two times five alternating) bundles in the pedicel (Figure 4A, cross Section 1). Thereof, one set of five bundles forms the sepal supply (Figure 4A,B, green) with one each becoming a sepal midrib bundle. The second alternating set of five bundles (Figure 4A,B, grey) differentiates into the supply for petals (Figure 4A,B, red), stamens (Figure 4A,B, yellow), and carpels (Figure 4A,B, purple). Each bundle of this second bundle set splits into three bundles forming a ring of 15 bundles, grouped into five triplets alternating with the sepal midrib bundles (Figure 4A, cross Section 2; rectangle indicates one triplet). The middle bundle of each triplet forms the main petal supply (Figure 4A, cross Section 3), while each lateral bundle of a triplet fuses with the according lateral bundle from the neighboring triplet (Figure 4A, arrows in cross Section 2) to form a stamen bundle and also provide bundles running into sepals as secondary sepal bundles (Figure 4A,B, orange). Each of these secondary sepal bundles later splits into two resulting in a total of four secondary bundles per sepal (Figure 4A, cross Sections 3–5). Thus, each stamen bundle is formed by fusion of two adjacent bundles of different origin; the sepal supply is heterogenous as well, i.e., the secondary setal bundles do not branch from the main sepal bundle but are provided by neighboring bundles. Each main petal bundle later on forms two side branches, which split again further up resulting in one main and four lateral bundles per petal (Figure 4A,B, red). In the petal tips, these bundles ramify into a dense network of bundles. Each main stamen bundle later splits again (Figure 4A, cross Section 3) and each provides two thin bundles that run into the carpel wall (Figure 4A, cross Section 4, purple). These carpel bundles branch further resulting in two semicircles with 5–6 bundles each around the carpels. In addition, each carpel seems to be supplied by a main central bundle (Figure 4A, cross Section 5) for which the origin could not be retraced; both these main bundles fuse where the carpel tips are postgenitally fused. The smaller bundles surrounding each carpel in a semicircle merge into one ventral bundle per carpel, which both run into the style-head (cf. Figure 3E; see also [5]). Further up in the style-head, both these bundles again divide into smaller branching bundles (cf. Figure 3F). No vascular bundles could be detected in the corona and colleters, neither by micro-CT scanning nor by LM (Figure 4A,B and Figure 3B–E). A movie of a rotating 3D model of the vascular system in a mature trap flower of *C. sandersonii* can be found in the Supplementary Materials (Movie S1).

### 2.4. Phylogenetic Analyses of Ceropegia sandersonii MADS-box Genes

**Figure 5:**
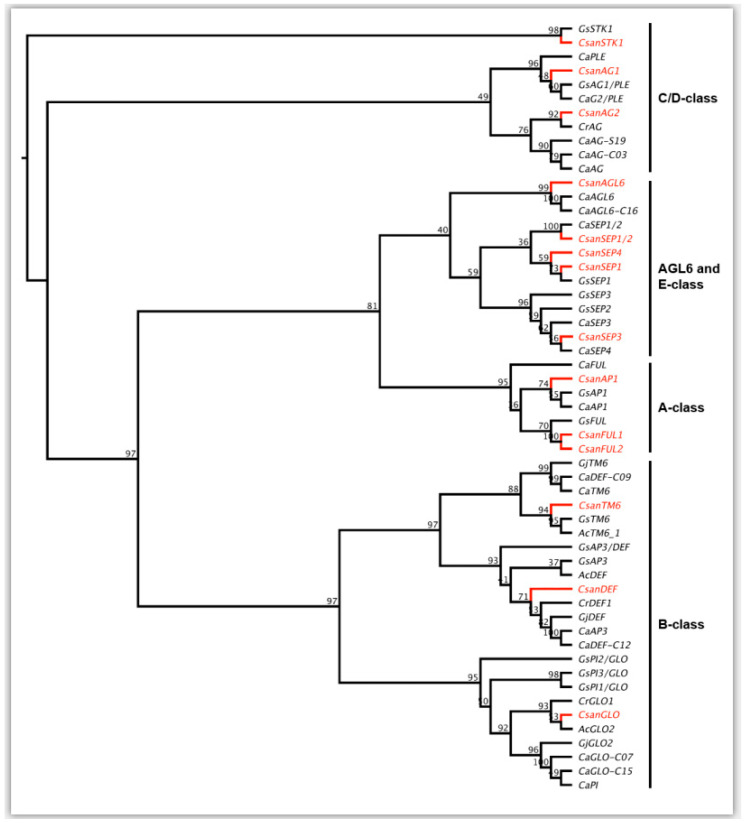
Maximum Likelihood tree of MADS-box lineages analyzed. Gene copies identified in our floral reference transcriptome of *Ceropegia sandersonii* (*Csan*) are indicated in red. Numbers above the nodes represent bootstrap values. Ingroup DNA sequences include data from taxa in Gentianales (Apocynaceae-Rauvolfioideae, Gentianaceae, Rubiaceae; obtained from NCBI GenBank; see Table S2) with the following abbreviations: Ac = *Allamanda cathartica*, Ca = *Coffea arabica*, Cr = *Catharanthus roseus*, Gj = *Gardenia jasminoides*, Gs = *Gentiana scabra. AG = AGAMOUS*; *AGL6* = *AGAMOUS-LIKE6*; *AP = APETALA*; *DEF = DEFICIENS*; *FUL* = *FRUITFULL*; *GLO = GLOBOSA*; *PI = PISTILLATA*; *PLE = PLENA*; *SEP = SEPALLATA*; *STK = SEEDSTICK*; *TM6 = TOMATO MADS BOX GENE6*.

A floral reference transcriptome was generated from the RNA-Seq data obtained from early buds and mature sepals, petals, and gynostegia samples of *Ceropegia sandersonii*. In this reference transcriptome, a total of 14 MADS-box gene copies belonging to the five main classes of MADS-box gene homologs known for Gentianales were identified (see Figure 5). MADS-box A-class genes were represented by one *APETALA1* gene (*CsanAP1*) and two *FRUITFUL* gene lineages *CsanFUL1* and *CsanFUL2,* which were found to be closely related to a *Gentiana scabra* homolog (*GsFUL*) in our phylogenetic analyses (Figure 5). For B-class genes, we identified one homolog of the *APETALA3*/*DEFICIENS* lineage (*CsanDEF*), one homolog of *TM6* (*CsanTM6*), and one *GLOBOSA* (*CsanGLO*) gene. The C-class genes *PLETHORA* and *AGAMOUS* were represented by the two homologs *CsanAG1* and *CsanAG2*, and from the D-class gene lineage *SEEDSTICK* one homolog (*CsanSTK*) was identified. E-class genes were represented by *AGAMOUS-LIKE6* (*CsanAGL6*), and four copies of *SEPALLATA* genes from four different clades (*CsanSEP1*, *CsanSEP1/2*, *CsanSEP3*, *CsanSEP4*).

### 2.5. Differential Gene Expression Analyses

**Figure 6:**
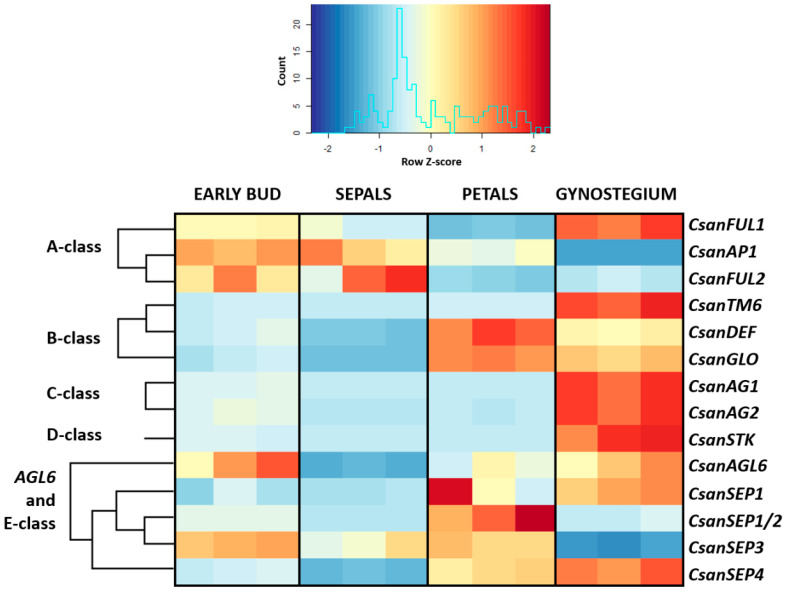
Heatmap visualizing expression differences (count numbers) of 14 MADS-box genes between early floral buds, mature sepals, petals, and gynostegia of *Ceropegia sandersonii*. Each tissue type was analyzed in three biological replicates. The color key of the heatmap is based on the number of counts matching the reference transcriptome divided by the total number of counts obtained for each sample type; cold colors indicate relatively low numbers and warm colors relatively high numbers of counts.

Differential gene expression analyses, as visualized in a heatmap (Figure 6), indicated clear differences in organ specific expression patterns of the 14 different MADS-box gene copies investigated. In early buds, *CsanFUL1*, *CsanAP1*, *CsanFUL2*, *CsanAGL6*, and *CsanSEP3* showed higher expression than all other genes in this developmental phase (Figure 6). Nine genes, i.e., *CsanTM6*, *CsanDEF*, *CsanGLO*, *CsanAG1*, *CsanAG2*, *CsanSTK*, *CsanSEP1*, *CsanSEP1/2*, and *CsanSEP4,* were higher expressed in mature flowers than in early floral buds (Figure 6). Overall, in mature flowers, MADS-box A-class genes were expressed in the gynostegium samples, in the sepals, and in the petals. MADS-box B-class genes showed high expression in the gynostegium and petals. MADS-box C- and D-class genes were highly expressed in the gynostegium and MADS-box E-class genes were expressed in all tissue types, however, with copy specific differences (Figure 6).

### 2.6. RT-PCR Experiments with Selected MADS-Box Genes

**Figure 7:**
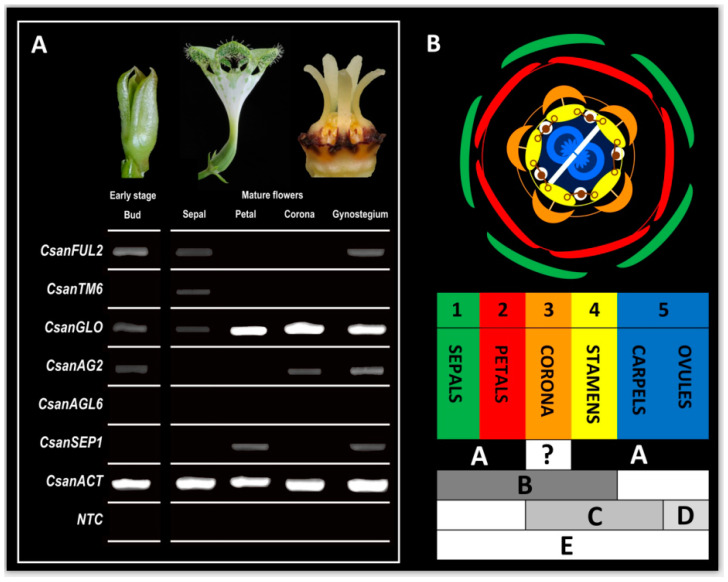
Expression patterns (RT-PCR) and ABCDE model of MADS-box gene expression in *Ceropegia sandersonii*. (**A**) Expression profiles of MADS-box gene homologues *CsanFUL2*, *CsanTM6*, *CsanGLO*, *CsanSEP1*, *CsanAG2*, *CsanAGL6* in early floral buds, mature sepals, petals, corona, and gynostegium. Results are based on three RT-PCR replicates per gene and tissue type. Ubiquitously expressed ACTIN (*CsanACT*) was used as a positive control. NTC = no template control; (**B**) floral diagram of *C. sandersonii* pitfall flowers (top) and model for expression of MADS-box gene classes involved in floral organ development (bottom). In the floral diagram (after [5]), fusion events within and between whorls are indicated by lines. Congenital fusion occurs among petals (red), corona lobes (orange), stamens (yellow), and between corona lobes and stamens. Postgenital fusion occurs between stamens and the style-head (light blue), and between the two carpels (dark blue). MADS-box E-class genes are expressed in all floral organs. Combined MADS-box A- and B-class genes control formation of sepals (green) and petals (red); combined MADS-box A-, B-, and C-class genes are involved in the formation of stamens (yellow); combined expression of MADS-box A- and C-class genes forms the carpels (blue), and combined MADS-box A- and D-class gene expression leads to the formation of ovules. The formation of the corona is controlled by combined MADS-box B- and C-class genes, indicating a staminal origin of this highly specialized organ, placed between the stamens and the petals. Photographs by A.H. and David Styles.

The differential expression patterns indicated by our transcriptome analyses (see above) were investigated in more detail for the six MADS-box gene homologs *CsanFUL2*, *CsanTM6*, *CsanGLO*, *CsanAG2*, *CsanAGL6*, and *CsanSEP1*, using RT-PCR. Again, these MADS-box gene homologs showed distinct expression patterns between the investigated sample types, i.e., early-stage floral buds, and mature sepals, petals, gynostegia, and coronas (Figure 7A). However, the results of the RT-PCR analyses were partly different from what was previously indicated in the transcriptome analyses (RNA-Seq), which we performed for these six genes (Figure 6); only *CsanTM6* showed a significant positive correlation between the RNA-Seq results and those obtained by RT-PCR (see Figure S3). In the RT-PCR experiments, *CsanFUL2* was found to be expressed in early floral buds, and in sepals and gynostegia of mature flowers. *CsanTM6* was only found to be active in mature sepals. *CsanGLO* was expressed in all tissue types analyzed, *CsanAG2* was active in early floral buds, and mature gynostegia and corona tissue. *CsanSEP1* was expressed in mature petals and gynostegia, whereas *CsanAGL6* was not expressed in any of the tissues analyzed.

## 3. Discussion

### 3.1. Development and Synorganization of the Highly Specialized Corolla and Corona

The peculiar parachute-like morphology of *Ceropegia sandersonii* trap flowers is due to unusual postgenital fusion of the corolla lobe tips into an umbrella-like structure above the corolla tube (Figure 1A; see also [21]). In a few other species, such as *C. rendallii*, *C. fimbriata*, *C. galeata*, *C. huberi*, and *C. connivens*, the corolla lobe tips also form an umbrella though less marked than in *C. sandersonii* and with a different development (see [21] for comparison to *C. rendallii*). Furthermore, the uvula, a dark purple protuberance on the underside of the umbrella (Figure 1A,B), is unique to *C. sandersonii* [21]. In *Ceropegia*, the corolla lobe tips comprise osmophoric tissue (see [18,29]) from which floral scent is emitted to attract fly pollinators. In *C. sandersonii*, osmophoric tissue is arranged in specific zones on the umbrella-like flower tip (Figure 1C). These osmophoric zones can be identified by the papillate epidermis (Figure 1E) also found in other *Ceropegia* species (e.g., *C. thaithongiae* [30]; *C. stapeliiformis* and *C. elegans* [29]). The osmophoric zones alternate with gliding zones (Figure 1C; see also [18,21]) comprised of intriguingly acuminate to caudate cells (Figure 1D; see also [18]), which clearly differ from the papillate epidermis cells found in osmophores of *Ceropegia*; the uvula is part of these gliding zones (Figure 1C). Previous studies described the epidermis of gliding zones to be covered by waxy secretions [18,21] which promote trapping of fly-pollinators as they lose foothold on this slippery tissue surface. Attracted flies which walk onto the gliding zones were indeed observed to slip off and fall into the funnel-shaped corolla throat. We did not discern waxy secretions nor residuals thereof on the corolla lobes of *C. sandersonii*; however, the spikey cells of the gliding zones secreted droplets of a translucent liquid that did not appear to be of a waxy nature. The finding that these epidermal cells stained intensively with neutral red indicates high permeability and their secretory nature. Flower-visiting flies were observed to eagerly probe the epidermis surface with their probosces (see also [23]), which suggests that they feed on the liquid droplets. The acuminate nature of the cells presumably reduces the surface the flies’ tarsi can hold onto so that they lose grip and slip off. Once a fly is trapped inside the inflation of the corolla tube, pollinarium removal and pollinium insertion, respectively, are achieved by correct positioning of a trapped fly at the gynostegium [31]. For this particular part of the pollination process, the corona around the gynostegium plays a decisive role to achieve attachment of pollinaria to a fly’s mouthparts and insertion of a pollinium between the guiderails, respectively.

The elaborate trap flowers of *Ceropegia sandersonii* develop in 10 phases (P1–P10, Figure 2A–P) in which clear changes in organ formation take place. During these phases, the primordia of different organs are initiated in a sequence from outer to inner whorl, i.e., sepals are initiated first, followed by petals, then androecium, and gynoecium; the corona is initiated last between petals and androecium, as is considered normal for this plant group (see [8]). The five sepals appear and develop one after the other in a particular ontogenetic sequence (see Figure 2A,B) found in other plant groups as well (see [8]). The first and the second developing sepal, as well as the third and fourth sepals, are approximately opposed, and the fifth sepal develops between the second and the third. This ontogenetic sequence allows for optimized protective coverage of the inner organs [8], which develop simultaneously per whorl. As soon as all sepals have reached a certain width, they show normal quincuncial position.

In *Ceropegia sandersonii*, the corona was found to only appear in developmental phase P7 and after all other organs reached a certain degree of development, conforming what was described previously for related taxa [5,14,32]. The corona structure differs between *Ceropegia* species and it acts as a morphological filter to channel pollinator specificity, resulting in reproductive isolation and, ultimately, speciation. Thus, coronas have received much attention in comparative anatomical investigations (e.g., [10,13,14,33]). There are generally two types of coronas, i.e., corolline and gynostegial coronas. The evolutionary developmental origin of corolline coronas is petal tissue, whereas the gynostegial coronas derive from stamen tissue, with a distinction between staminal coronas and interstaminal coronas (see [10,13]). Within Asclepiadoideae, the staminal corona was described as a key innovation and found to be ancestral in this subfamily [10]. We combined anatomical, developmental, and molecular methods to explore the evolutionary origin of the corona in *Ceropegia sandersonii* and clearly determined it as being of staminal origin. Anatomical and developmental investigations showed that it emerges from the base of the stamens with one lobe each appearing dorsally along a stamen. A similar corona initiation was described and illustrated in *C. haygarthii* using SEM analyses [8]. In *C. attenuata*, the corona was described to emerge from the staminal column [15], as found based on microtome sectioning; however, SEM images to compare the point of initiation are not available for this species or for any other *Ceropegia* species. The morphological and structural diversity of coronas in *Ceropegia* allow to assume differences regarding initiation and ontogeny, and further SEM based studies should be carried out to assess such (dis)similarities. In general, stamen derived coronas seem to appear much later during floral ontogeny than corolline coronas, which were found to be initiated already once the petals start to postgenitally fuse [10].

### 3.2. Con- and Postgenital Fusion of Floral Organs

*Ceropegia* flowers show high degrees of synorganization via con- and postgenital fusion of floral organs within and between floral whorls. These fusion events were previously described merely as either ‘early’ or ‘late’ during floral ontogeny [5,8]. The definition of 10 phases during ontogeny of *Ceropegia sandersonii* trap flowers presented here allows a much more detailed timing of these different types of fusion events, as well as their relative contribution. Congenital fusion events occur during phases P3 (petal bases; Figure 2C), P4 (stamens; Figure 2E), and P7 (between stamen and corona; Figure 2M). Postgenital fusion takes place in P4 and P7. In P4, the petal tips start to postgenitally fuse, forming pouches at the petal sinuses (Figure 2D). These pouches are a typical sign for postgenital fusion [10] and disappear later on when the petal tip margins are entirely postgenitally fused (Figure 2F). In P7, the carpel tips are postgenitally fused and as a result the style-head develops into a pentagonal structure with five glands (Figure 2N, O; see also Figure 3D–F). Furthermore, in P7, the style-head postgenitally connects with the anthers, which is a prerequisite for the formation of the pollinaria (see [5,8]).

### 3.3. Vascularization of Ceropegia Pitfall Flowers

The positions and connections of vascular bundles are traditionally studied using microtome slicing and light microscopic investigation of selected sections. The subsequent description of a vascular system is based on few selected sections and line drawings thereof. The disadvantage of this conventional approach is that the entire vascular system can hardly be visualized in its original three-dimensional structure. Studying vascularization using micro-CT scanning of flowers in combination with light microscopy provides a more informative insight. The resulting three-dimensional image of the entire vascular system can be rotated and studied from all possible perspectives to identify the origins of tissue specific vascular bundles especially in early developmental stages. 3D data facilitate comparative analyses and conclusions on the evolutionary origin of floral organs of unclear homologies. Combining the study of both microtome sections and 3D-images allow a comprehensive understanding with realistic visualization of floral vascularization. Novel 3D X-ray micro-computer tomography techniques (micro-CT) proved to be a powerful tool to study vascularization of orchid flowers (e.g., *Erycina pusilla* [34]; *Phalaenopsis equestris* [35]). To the best of our knowledge, this method has not yet been applied to any species in the Gentianales before. Aspects of floral vascularization were studied in a subset of species in Apocynaceae [15,36], including a single *Ceropegia* species, i.e., *C. attenuata*, using conventional techniques. The origin of vascular bundles was described as being basically similar to that of species in the related genera *Cynanchum*, *Sarcostemma*, *Tylophora*, and *Pentatropis* [15]. However, the description and line drawings do not provide much detail. We aimed to provide a basis for more straightforward comparative studies on vascularization in related taxa using 3D scans.

We applied micro-CT scanning to visualize the vascular system in mature *Ceropegia sandersonii* pitfall flowers and provide a 3D-model of vascular bundles. Our micro-CT scans allowed to reveal similarities but also differences and new insights into how floral organs are connected via vascular bundles in Apocynaceae [15,36]. In *C. sandersonii*, each stamen is supported by a single vascular bundle that runs all the way through this floral organ without branching, similar to other Apocynaceae [36]. We found that stamen bundles are formed by fusion of veins with separate origin, i.e., bundles that derive from two neighboring main bundles (Figure 4A, cross Section 2; Figure 4B-4); a double origin of stamen bundles was also described and discussed in other plants [37,38]. Vascular bundles running into the sepals are of mixed origin as well, i.e., one main bundle is joined by secondary bundles originating from an adjacent bundle, which also provides stamen and petal supply (Figure 4B-3). Carpels were found to be fed by vascular bundles originating from stamen bundles (Figure 4A). The androecium and gynoecium together form the gynostegium and thus a shared vascular supply seems logical at first sight. However, synorganization between style-head (gynoecium) and anthers (androecium) happens via postgenital fusion, which makes the presence of shared vascular bundles impossible. This aspect should be further addressed in follow up studies.

Generally, the first and main vascular bundle of a floral organ is initiated by the corresponding organ in its youngest developmental stage (see [39] and references therein). This isolated early stage bundle very rapidly develops downward and connects with the closest bundle in a similar developmental stage. It then also develops upwards during further organ growth. LM analyses of early flower buds suggest a similar bundle development in *Ceropegia sandersonii* trap flowers. The exact time points of bundle initiation and fusion events are to be clarified in a detailed study focusing on vascular bundle development in very early stages of flower development; 3D micro-CT scans could facilitate such a study.

We did not discern vascular bundles in the staminal corona of *Ceropegia sandersonii*, neither with micro-CT nor with LM methods. Stamen derived corona structures generally seem to be non-vascular [15], and the absence of any vascular bundles in the corona of *C. sandersonii* supports an entirely stamen derived origin of this organ. Most species of Apocynaceae–Asclepiadoideae offer considerable amounts of nectar in coronal nectar cups. In deceptive *Ceropegia* species, the presence of nectar in the interstaminal coronal cavities is uncertain, and if present at all, then in only very little amounts (see [40]). It is furthermore unclear where the nectar that accumulates in the coronal cavities is produced. It has been suggested that the stigmatic chambers behind the guide rails are nectariferous [40], as described in other Asclepiadoideae [32]. In *C. sandersonii*, the presence of nectar was never empirically verified but the absence of vascular bundles in the corona support the assumption that in *Ceropegia* the corona tissue itself is not nectariferous but only acts as a presenting device for nectar produced by a primary nectary [41]. However, staminal coronas with nectariferous epidermal cells have been found in Asclepiadoideae, e.g., in *Gonolobus denticulatus* (as *Matelea denticulata*), *Peplonia axillaris,* and *Vincetoxicum hirundinaria* (as *Cynanchum vincetoxicum*) [32,42], but interestingly these coronas are not vascularized either [15,36]. In taxa with corolline coronas, e.g., *Cryptostegia* [10], the corona is vascularized by bundles originating from the petals [36]. In species with both corolline and staminal coronas, e.g., in *Gymnema* [10,13], vascular bundles originating from the petals were found to feed the corona [15].

### 3.4. MADS-Box Genes Driving Floral Organ Identity

With the transcriptome analyses performed in the present study, we aimed to obtain a floral reference transcriptome of *Ceropegia sandersonii* and for the first time identify MADS-box genes involved in floral organ formation of *Ceropegia* trap flowers. The detection of isoforms, co-expression network analysis, and metabolic pathway reconstructions were beyond the scope of the current study. The floral reference transcriptome of *C. sandersonii* was generated from RNA-Seq data of early buds, and mature sepals, petals, and gynostegia. A total of 14 MADS-box genes were identified in the transcriptome, with representatives of all major MADS-box gene classes. RNA-Seq analyses indicated these genes to be differentially expressed between the different tissue types investigated, i.e., early buds, and mature sepals, petals, and gynostegia (Figure 6). Semi-quantitative RT-PCR methods were applied to validate the differential expression for six selected MADS-box genes (*CsanFUL2*, *CsanTM6*, *CsanGLO*, *CsanAG2*, *CsanAGL6*, and *CsanSEP1*) in these tissue types plus in the corona of mature flowers. The results obtained from both RNA-Seq and RT-PCR analyses were combined for the development of a modified ABCDE-model for MADS-box genes involved in organ identity of highly synorganized *Ceropegia* pitfall flowers (Figure 7B). According to this model, A-class gene expression leads to formation of sepals, petals, and gynostegia. B-class genes are expressed in mature sepals, petals, stamens, and the corona but not in the gynoecium (carpels and ovules). C-class genes are expressed in the mature corona, stamens, and carpels but not in the ovules. D-class genes are only active in ripening ovules, and E-class genes are expressed in all floral organs. In our proposed ABCDE-model, the MADS-box gene classes involved in the formation of stamens, carpels, and ovules are inferred from models of other flowering plants see [43] as we did not separate these tissue types but used complete gynostegia in our transcriptome analyses. The MADS-box gene expression in *C. sandersonii* stamens, carpels, and ovules hypothesized here remains to be tested.

In our reference transcriptome of *Ceropegia sandersonii* flowers obtained from early and mature floral tissue, three A-class genes were identified, i.e., two copies of *FRUITFULL* (*FUL*) and one copy of *APETALA1* (*AP1*). Our expression analyses confirm that A-class genes play a role in formation of both early floral tissue and mature floral organs (see Figure 6) as found for many other plant taxa [44].

B-class genes generally control identity of petals and stamens. In our expression analyses, the *GLOBOSA* homolog *CsanGLO* was found to be expressed in all investigated floral organs, both in early and mature stage, which indicates a key function of this B-class gene in development of *Ceropegia* pitfall flowers, in line with the floral quartet model [43,45].

Exploring the evolutionary origin of the corona in *Ceropegia* was of special interest in the present study. Due to the exceptional diversity of corona morphology and development (petal or/and stamen derived, i.e., corolline or/and staminal) within Apocynaceae, the evolutionary origin and homology of this floral organ is not yet fully understood. Based on previous ontogenetic studies with traditional anatomical methods, the corona was assumed to be corolline in Rauvolfioideae, Apocynoideae, and Periplocoideae, but typically of staminal origin in Asclepiadoideae and Secamonoideae [10,13]. In *C. sandersonii* (Asclepiadoideae), the B-class gene *GLOBOSA* (*CsanGLO*) was found to be expressed in the early and mature corona together with the C-class gene *AGAMOUS* (*CsanAG2*). Combined MADS-box B- and C-class gene expression, as well as the results of our SEM and micro-CT analyses support the idea that in *Ceropegia* the corona is of the ancestral staminal type described as an evolutionary key invention in Asclepiadoideae [10].

Synorganization between androecium and gynoecium is also found in Orchidaceae and *Aristolochia* (Aristolochiaceae) where the resulting structure is called a gynostemium. In orchids, specialized stamen derived structures (lateral projections at the upper part of the column) on both sides of the gynostemium, i.e., the stelidia, promote correct positioning of pollinators at the gynostemium, similar to the function of the corona in Apocynaceae. In these stelidia, combined MADS-box B- and C-class gene expression was also found [34].

No E-class gene expression was found in the mature corona of *Ceropegia sandersonii*. In the callus on the orchid labellum, also an organ of staminal identity [34,35], E-class gene expression was found. Expression was more pronounced in early than in late developmental stages of the callus and thus, E-class gene expression may take place in the early stage *Ceropegia* corona as well. This aspect, however, could not be assessed in this study because early-stage coronas were too small to dissect for RNA extractions. Laser capture microdissection could be applied for further investigation.

E-class gene expression in *Ceropegia* was restricted to mature petals and gynostegia. This finding is congruent with studies in *Coffea* [46], the closest relative of *Ceropegia* for which MADS-box gene expression was analyzed, where no E-class gene expression was detected in young floral buds either.

## 4. Materials and Methods

### 4.1. Plant Material

*Ceropegia sandersonii* is a succulent climber from South Africa (see [47]). Its bright green and white flowers have a peculiar parachute shape (see Figure 1A), which is why this species is commonly known as “Parachute Plant”. For our study, we cultivated 12 different individuals (purchased from Paul Shirley Succulents, https://www.paulshirleysucculents.nl/) in the Hortus botanicus Leiden, The Netherlands. Voucher specimens (see Table S1) of the study plants are deposited in the herbarium of Naturalis Biodiversity Center.

### 4.2. Fixation of Flowers for Micromorphology (micro-CT, SEM)

Fresh mature flowers and buds were harvested in different stages and fixed with standard formalin-aceto-alcohol (FAA: Ethanol absolute, 90%; glacial acetic acid, 5%; formalin 5%). The samples were stored at room temperature until further use.

### 4.3. Scanning Electron Microscopy (SEM)

Floral buds at different developmental stages were dissected in 70% ethanol and subsequently washed twice each with 70% and 96% ethanol. The material was then transferred to 100% acetone, which was changed after 30 min. Subsequently, the samples were critical point dried using liquid CO_2_ with a Leica EM CPD300 critical point dryer (Leica Microsystems, Wetzlar, Germany), and mounted on aluminum stubs using either double-sided carbon tape or Leit-C carbon cement. A Quorum Q150TS sputter coater (Quorum Technologies, Laughton, East Sussex, UK) was used to coat the samples with a 20 nm thin layer of Platina-Palladium. Imaging of samples was performed with a JEOL JSM-7600 F Field Emission Scanning Electron Microscope (JEOL Ltd., Tokyo, Japan).

### 4.4. Light Microscopy (LM)

To further investigate the morphology of epidermal surfaces in *Ceropegia sandersonii*, mature flowers were harvested from life plants, and freshly dissected tissue as well as tissue stained with neutral red (1:10000 neutral red:tap water; for 2–10 h) was investigated under a Light Microscope. Light Microscopy was also applied to further investigate vascularization and organ development in *C. sandersonii* flowers. Therefore, early and late floral buds were harvested from plants and fixated in 70% ethanol for seven days. Buds were then stepwise dehydrated in ethanol (80%, 96%) over three days and stored in ethanol absolute for five days. Subsequently, buds were infiltrated with xylol by increasing the concentration of xylol (ethanol:xylol ratios: 2:1, 1:1, 1:2) over three days and stored in 100% xylol for three days. Xylol was then gradually substituted with paraffin (KP Paraclean I) over a period of three days (xylol:Paraclean ratios: 3:1, 1:1, 1:2) at 60 °C, and buds were stored in 100% Paraclean for four days at 60 °C. Before embedding, any remaining air was pumped from the material in a vacuum desiccator (−1000 mBar) at 60 °C for 30 min. Samples were sliced using a Leica RM 2265 microtome equipped with a Leica DB 80 LX disposable blade, and at a thickness of 8 µm. Sections were placed on microscope slides and dried for one hour on a heat plate at 40 °C. For staining, sections were gradually deparaffinated. Therefore, they were kept in xylol for 7 min renewal of xylol after 5 min), then transferred to a mixture of ethanol and xylol (1:1) for 2 min, and gradually rehydrated in ethanol (96%, 70%, and 50%; 2 min each). The sections were then rinsed with demineralized water for 1min and stained with freshly made Etzold’s solution (Fuchsin, Safranin O, Astra blue) for 2 h. After staining, the slices were rinsed with tap water and demineralized water for 1 min and 15 s, respectively, again gradually dehydrated in ethanol (50%, 70%, and 96%; 15 s each), transferred to ethanol:xylol (1:1) for 2 min, and finally to 100% xylol. The slices were mounted with DPX-new-100579 (Merck Chemicals B.V., Amsterdam, NL). Images were taken using a Zeiss AxioVision stacking microscope (Carl Zeiss Microscopy GmbH., Jena, Germany).

### 4.5. 3D X-ray Micro-Computer Tomography (micro-CT)

Fresh mature flowers were stained for 5 days with 1% phosphotungstic acid (PTA) in 70% ethanol as contrast agent whereby PTA was changed daily. After staining, flowers were washed twice with 70% ethanol and embedded in 1.5% low melting point agarose in a plastic container. Embedded flowers were scanned using a Zeiss Xradia 510 Versa 3D X-ray equipped with a sealed transmission X-ray source (settings: voltage/power: 40 kV/3 W; source current: 75 μA; exposure time: 2 s; camera binning 2; optical magnification: 4x; pixel size: 3.5 μm; total exposure time: ~2–3 h). Single 2D images were stacked to build a 3D image, which was processed using Avizo 3D software version 8.01 (Thermo Fisher Scientific, Waltham, MA, USA).

### 4.6. RNA Isolation

RNA was isolated from floral tissue of *Ceropegia sandersonii* to produce a reference transcriptome for identification of MADS-box genes, and for further RT-PCR analyses (see below) with selected MADS-box genes. Therefore, early floral buds (<3 mm) and mature flowers (first day of anthesis) were harvested from different plant individuals. Mature flowers were dissected to sepals, petals (tip, tube, base), gynostegium, and corona. The tissue was transferred into sterile 2.2 mL microcentrifuge tubes, together with a glass bead (7 mm; Assistent). For each sample type, tissue was dissected and pooled from 3–6 flowers per plant individual (i.e., 3–5 early buds, sepals from 2 flowers, gynostegia from 3–5 flowers, and corona tissue from 5–6 flowers), to reach the required amount of tissue needed for a sufficient RNA yield (min. 30 mg, max. 90 mg). For petals, the amount of tissue from a complete petal (base to tip) exceeded the maximum amount of tissue; thus, each petal was dissected into tip, tube, and base, and RNA extracted from these subsamples was again pooled to obtain a complete petal sample. All samples were snap-frozen in liquid nitrogen immediately after harvesting and stored at −80 °C until RNA isolation. The frozen plant tissue was ground three times á 15 s using a TissueLyser II (Qiagen Benelux BV, Venlo, The Netherlands); each round was followed by re-freezing of samples in liquid nitrogen. Total RNA was extracted using the RNeasy Plant Mini Kit (Qiagen Benelux BV, Venlo, The Netherlands) under RNAse free conditions. The protocol was adapted by including a step to digest single and double stranded DNA (DNase I; Amp Grade, Invitrogen 1U/µL). The quality and quantity of RNA per sample was measured using NanoDrop (ND-1000 Spectrophotometer, Marshall Scientific). Samples selected for RNA sequencing were further quality checked by determining the integrity (RNA Integrity Number; RIN) using the Plant RNA nano protocol on an Agilent 2100 Bioanalyzer (Agilent Technologies). RNA samples of early buds and mature sepals, petals, and gynostegia with a RIN >9.5 were used for NGS sequencing. Samples of corona tissue did not yield in sufficient high-quality RNA for NGS sequencing; but this sample type could be used for downstream RT-PCR analyses (see below). RNA samples of early buds and mature sepals, petals, and gynostegia were prepared in three biological replicates each (from three different plant individuals) and the 12 samples were sent to the Beijing Genomics Institute (BGI) for de novo NGS sequencing on an Illumina HiSeq platform (PE150). An mRNA library (>30MR per sample) was constructed (for details see Text S1), and sequencing was performed with a HiSeqTen instrument reading 150 bases paired-end reads.

### 4.7. Transcriptome Analyses and MADS-Box Gene Identification

After NGS transcriptome sequencing, the generated and cleaned reads (fastq format) were downloaded from the BGI cloud server. Further analyses were performed using an in-house designed bioinformatic pipeline (Naturalis OpenStack server, accessed via PuTTY, https://www.putty.org/) for quality control, assembly, annotation, and differential expression analysis (https://github.com/naturalis/orchid-transcriptome-pipeline/tree/master/Scripts).

Cleaned read pairs provided by BGI were quality checked using FastQC v0.10.1 (http://www.bioinformatics.babraham.ac.uk/projects/fastqc). Low-quality reads as well as reads with insufficient coverage were trimmed or removed using Trimmomatic v0.32 [48] (max. mismatch count: ‘2′, accuracy of match between two adapter ligated reads: ‘30′, accuracy of match between any adapter: ‘10′, *LEADING*: ‘3′, *TRAILING*: ‘3′, *SLIDINGWINDOW*: ‘4:20′, *MINLEN*: ‘50′); adapter remnants were removed using the *ILLUMINACLIP* option; remaining reads were again quality checked. The FastQC quality reports are provided in the Supplementary Materials (Folder S1). Trinity v2.5.1 ([49]; parameters: Default) was used for de novo assembly of cleaned reads without a reference genome (paired-end reads setting *PE*). CDHIT-EST [50] was used to cluster the produced contigs into longer transcripts (parameters: −n ‘9′, −d ‘0′, −M ‘0′, −T ‘20′) and to create consensus sequences. Reads were aligned back against the generated de novo reference transcriptome using RSEM v1.3.0 [51] which internally uses Bowtie2 v2-2.3.3.1 [52], to produce SAM files. Raw counts per read were quantified and a count table was generated using RSEM (function: *rsem-generate-data-matrix*). The contig metrics are as follows: Total assembled bases: 404,715,268; total number of contigs (>201 bp): 298466; median contig length: 406 bp; average contig length: 724 bp; largest contig: 15689 bp; contig N50: 1174 bp; contig GC%: 42.01%. The transcriptome data (RNA-Seq libraries) generated in the present project were uploaded to NCBI (BioProject accession number: PRJNA678862; https://www.ncbi.nlm.nih.gov/sra/PRJNA678862).

To specifically identify and annotate MADS-box genes in the generated reference transcriptome, all *Ceropegia sandersonii* gene sequences in the transcriptome were blasted against a local database of Gentianales MADS-box gene homologs (*Rubiaceae: Coffea arabica*, *Gardenia jasminoides*; Gentianaceae: *Gentiana scabra*; *Apocynaceae: Allamanda cathartica*, *Catharanthus roseus*; see Table S2), which was created by retrieving DNA sequences from NCBI GenBank. Sequences for Actin were also retrieved to identify the *C. sandersonii* actin homolog required as control gene for the RT-PCR experiments (see below). 4.8. Phylogeny of MADS-box gene lineages.

To assess phylogenetic relationships between Gentianales MADS-box gene lineages, all publicly available DNA sequences (see Table S2) plus the newly identified *Ceropegia sandersonii* MADS-box gene homologs (see Table S1) were translated to amino acids in the correct translation frame by using translate-protein tools (http://reverse-complement.com/translate-protein/ROOT/), loaded into Geneious Prime^®^ 2019 v2.3 (www.geneious.com), and aligned with the best matching open reading frame (from start to stop codon) using the ‘Geneious alignment’ function. The created alignment was trimmed down to the most conserved regions (protein domains and amino acid motifs) to ensure all sequences had similar length; regions that did not align were removed prior to further analysis. Separate alignments were made for each MADS-box gene subfamily and combined in a Maximum Likelihood phylogenetic analysis using the PhyML plugin [53] with the following settings: Substitution model ‘Blosum62′; Bootstrap ‘100′; proportion of invariable sites ‘0, fixed’; number of substitution rate categories ‘4′; gamma distribution parameter ‘0, estimated’; optimize ‘Topology/length/rate’; topology search ‘NNI (default, fast)’. As an outgroup, sequences that did not fall into the major subfamily clades were chosen, i.e., *SEEDSTICK* sequences of *Gentiana scabra* (*GsSTK1*) and the according homolog of *C. sandersonii* (*CsanSTK1*).

### 4.8. Differential Expression Analyses of Identified MADS-Box Genes

Differential expression analyses were performed for the 14 MADS-box gene homologs (see Table S1) identified in our *Ceropegia sandersonii* floral reference transcriptome. Expression differences between sample types and among the three biological replicates per sample type were visualized in a heatmap (Figure 6). The heatmap was generated based on the count table generated for the corresponding gene sequences (see above) using an in-house designed bioinformatic script (https://github.com/naturalis/orchid-transcriptome-pipeline/tree/master/Scripts). With this script, the number of matches between a specific read in the transcriptome and a reference gene was scored. Additional differential expression analyses were carried out using DESeq2 in RStudio v1.2.5033 [54] to calculate the log2 fold change of expression of the genes investigated in the different floral organs and developmental phases. Six pairwise tests between the four sample types ‘early buds’ and mature ‘sepals’, ‘petals’, and ‘gynostegium’ were performed (see Table S3). These tests identified those genes with significant differential expression (minimum log2 fold change of 0.25) between a given pair of sample types. All samples had >100 counts so that a cut-off for the analyses was not necessary. To visualize the DESeq2 results, a Principal Component Analysis (PCA) plot (Figure S1) as well as log ratio and mean average MA-plots (Figure S2) were generated.

### 4.9. Primer Design, cDNA Synthesis, and Semi-Quantitative Reverse Transcription PCR (RT-PCR)

To further investigate the differential MADS-box gene expression patterns indicated in the prior performed heatmap analyses (see above; Figure 6), six MADS-box gene homologs (*CsanFUL2*, *CsanTM6*, *CsanGLO*, *CsanAG2*, *CsanAGL6*, *CsanSEP1*) were chosen for further downstream analyses using semi-quantitative RT-PCR.

For this purpose, primers were designed for these six selected MADS-box genes using the online software Primer3Plus (https://primer3plus.com/cgi-bin/dev/primer3plus.cgi; settings: max. Poly-X = 3; CG-Clamp = 2; max. End GC = 2). All primer pairs (see Table S4) were screened for specificity in a gradient PCR reaction with a reaction mixture (25 μL) containing 10x CoralLoad Buffer (Qiagen), 25 mM MgCl_2_ (Qiagen), 100 mM Bovine Serum Albumin, Acetylated-BSA (Promega), 1.25x DMSO (Qiagen), 5x Q-Solution (Qiagen), 0.2 μM of each primer (IDT), 2.5 mM dNTPs (Qiagen), and 1.25 units/50 µL DNA *Taq* Polymerase (Qiagen), plus 100 ng cDNA template. MQ water (Ultrapure) was used to reach the final volume of 25 µL. The amplification started with an initial denaturation step of 6 min at 94 °C, continued with 38 cycles of three steps consisting of 1 min at 94 °C, 1 min at 52–55 °C, and 2 min at 72 °C, and was finalized with one amplification step of 12 min at 72 °C.

In addition to the sample types used for the RNA-Seq analyses (i.e., early buds and mature sepals, petals, and gynostegia), RT-PCR analyses were also performed with corona tissue dissected from mature gynostegia. RNA was isolated (as described above) from early floral buds, and from sepals, petals, gynostegia, and corona tissue of mature flowers, and cDNA was synthesized using SuperScript III reverse transcriptase (Invitrogen). As a first step, the reaction mix containing the RNA template (10 pg–5 µg), 1 µL 10 mM dNTP Mix, 50 µM Oligo (dT)_20_, and sterile distilled water (final volume: 14 µL) was heated at 65 °C for 5 min. After incubation on ice for at least one minute, the mix was briefly centrifuged, then 5X first-stand buffer (4 µL), 0.1 MDTT (1 µL), and 200 units/µL Superscript III (1 µL) were added. This mixture was then incubated at 55 °C for 50 min to dissolve the RNA template while avoiding formation of secondary structures; heating at 70 °C for 15 min inactivated the reaction. A reaction mix without RNA template but MQ water was used as negative control (non-template control, NTC). Quantity and quality of cDNA were measured via nanodrop (ND-1000 Spectrophotometer, Marshall Scientific). A total of 90 ng per cDNA sample was then used for PCR-amplification with sequence specific primers (see below). The Actin gene homolog (*CsanACT*) was used as positive control and a non-template reaction DNA (NTC) was used as negative control.

The RT-PCR analyses were performed in three replicates per gene and sample type. The thermal cycling regime used in the RT-PCR reaction was similar as for the gradient PCR (see above); however, the annealing temperature was set to 52 °C as this temperature yielded the best results and most specific products in the gradient PCR. Actin was amplified as a positive control; the negative control was a non-template control (NTC). All reactions were carried out in a CFX384 Touch Real-Time PCR system thermocycler (Biorad). The PCR products were run on a 1% agarose gel with 1x TAE and a 1 kb plus GeneRuler^TM^ (Thermo Scientific) as ladder. The gel was stained with ethidium bromide and digitally photographed using a gel doc (Ultima 10si, Isogen Life Science).

The differential expression patterns found for *CsanFUL2*, *CsanTM6*, *CsanGLO*, *CsanAG2*, *CsanAGL6*, and *CsanSEP1* using semi-quantitative RT-PCR were correlated to those found using quantitative RNA-Seq analyses. For this purpose, the absolute numbers of reads per gene obtained from RNA-Seq were converted into Transcripts Per Kilobase Million (TPM) values, which were then normalized by log2 transformation. The semi-quantitative RT-PCR results were quantified by scoring amplicons observed in the gels as present (1) or absent (0). The binary RT-PCR data were then correlated (Pearson correlation) with the transformed RNA-Seq data using the ggpubr package in RStudio v1.2.5033 [54]. The generated graphs are provided as Supplementary Materials (Figure S3).

## 5. Conclusions

This study investigated in comprehensive detail the development of highly complex and synorganized *Ceropegia sandersonii* pitfall flowers. We combined micro-morphological (LM, SEM, and micro-CT) and molecular techniques (transcriptome and RT-PCR analysis) to unravel floral organ development and identity. In developmental series from floral primordia to fully mature flowers, we identified 10 phases with distinct changes in floral organ development, in which con- and postgenital fusion occurred in 3 out of the 10. We furthermore performed the first transcriptome analyses of early buds and different tissues (sepals, petals, corona, gynostegium) of mature *Ceropegia* pitfall flowers and determined MADS-box genes involved in floral organ identity. We determined the corona in *C. sandersonii*, and thus likely in other *Ceropegia* species, to be of the ancestral staminal type, previously identified as evolutionary key invention in Asclepiadoideae. This is the first time that the origin of the corona was further clarified using molecular methods in a species of Apocynaceae. The corona appears along the base of the stamens, is not vascularized, and emerges late during floral development in phase 7 out of 10. Together with the combined MADS-box B- and C-class gene expression found in the corona, this suggests a staminal origin. Based on these results, we propose a first MADS-box ABCDE model for *Ceropegia* trap flowers, summarizing the main molecular mechanisms driving diversification of highly specialized deceptive trap flowers in Apocynaceae-Asclepiadoideae. The next steps to further validate the here proposed ABCDE model are qPCR analyses with laser dissected early-stage organs, and in situ hybridization analyses, which both were beyond the scope of the present study.

## Supplementary Materials

The following are available online at https://www.mdpi.com/2223-7747/9/12/1767/s1. Table S1: List of *Ceropegia sandersonii* MADS-box gene sequences identified in the floral transcriptome analyses in this study. NCBI accession numbers and numbers of herbarium vouchers (lodged at Naturalis Biodiversity Center) are given as well, Table S2: List of MADS-box gene sequences of Gentianales obtained from NCBI GenBank and used for phylogenetic analyses, Table S3: Results of differential gene expression analyses using DESeq in R, Table S4: List of primers used for semi-quantitative RT-PCR analysis, Movie S1: Movie of a rotating 3D model of vascular bundles in a mature trap flower of *C. sandersonii* based on 3D X-ray micro-CT scanning. Figure S1: DESeq2 generated Principal Component Analysis (PCA) plot of (dis-)similarities in expression profiles among and between the different tissue types, Figure S2: DESeq2 generated MA-plots of gene expression differences between the six sample type pairs, Figure S3: Correlation analyses between RNA-Seq and RT-PCR results. Folder S1: FastQC quality reports.
