# Supplementary material for: Pitfall Flower Development and Organ Identity of Ceropegia sandersonii (Apocynaceae-Asclepiadoideae)
Source: Plants (Basel). 2020 Dec 14;9(12):1767. doi: 10.3390/plants9121767 (PMC7764971; doi:10.3390/plants9121767)
Supplement: Supplementary file 1 [file plants-09-01767-s001.zip › plants-1003419_Revision1_Supplementary Material/Text S1_Library and sequencing.docx]

**Supplementary Text S1**

After total RNA extraction and DNase I treatment, the mRNA was isolated by using magnetic beads with Oligo (dT). Fragmentation buffer was added to digest the mRNA into shorter fragments from which cDNA templates were synthesized. After purification, EB buffer was added for end reparation and single nucleotide A (adenine) addition, followed by an adapter ligation step. Quality control was performed on an Agilent 2100 Bioanalyzer, and fragment amplification was performed using an ABI StepOnePlus Real-Time PCR System. Library sequencing was performed using Illumina HiseqTen reading at least 30 million reads (MR) per sample. Paired-end sequencing was applied for each sample to generate a read length of approximately 150 bp.
