# Supplementary material for: Pitfall Flower Development and Organ Identity of Ceropegia sandersonii (Apocynaceae-Asclepiadoideae)
Source: Plants (Basel). 2020 Dec 14;9(12):1767. doi: 10.3390/plants9121767 (PMC7764971; doi:10.3390/plants9121767)
Supplement: Supplementary file 1 [file plants-09-01767-s001.zip › plants-1003419_Revision1_Supplementary Material/Folder S1_FastQC.zip!/FastQC/CerA21_forward_paired_fastqc.html]

CerA21\_forward\_paired.fq FastQC Report 

FastQC Report

Wed 13 Feb 2019  
CerA21\_forward\_paired.fq

## Summary

- Basic Statistics
- Per base sequence quality
- Per tile sequence quality
- Per sequence quality scores
- Per base sequence content
- Per sequence GC content
- Per base N content
- Sequence Length Distribution
- Sequence Duplication Levels
- Overrepresented sequences
- Adapter Content
- Kmer Content

## Basic Statistics

| Measure | Value |
| --- | --- |
| Filename | CerA21\_forward\_paired.fq |
| File type | Conventional base calls |
| Encoding | Sanger / Illumina 1.9 |
| Total Sequences | 21167042 |
| Sequences flagged as poor quality | 0 |
| Sequence length | 50-150 |
| %GC | 41 |

## Per base sequence quality

## Per tile sequence quality

## Per sequence quality scores

## Per base sequence content

## Per sequence GC content

## Per base N content

## Sequence Length Distribution

## Sequence Duplication Levels

## Overrepresented sequences

No overrepresented sequences

## Adapter Content

## Kmer Content

| Sequence | Count | PValue | Obs/Exp Max | Max Obs/Exp Position |
| --- | --- | --- | --- | --- |
| CGCGGGG | 555 | 0.0 | 24.302916 | 1 |
| CGCCCTT | 5040 | 0.0 | 14.64875 | 1 |
| CGCGTAT | 1335 | 0.0 | 12.762265 | 1 |
| CTCGCTA | 5790 | 0.0 | 12.383361 | 9 |
| CGGGCAT | 6090 | 0.0 | 12.006536 | 1 |
| CGCCCAT | 5995 | 0.0 | 11.367889 | 1 |
| AGATCGG | 14885 | 0.0 | 11.096845 | 140-144 |
| GGAAAAT | 51095 | 0.0 | 11.003847 | 1 |
| GCCGGGT | 3360 | 0.0 | 10.986563 | 1 |
| CGGGCAC | 3545 | 0.0 | 10.61347 | 1 |
| CGGGCCT | 2775 | 0.0 | 9.976987 | 1 |
| CGGGTAT | 4140 | 0.0 | 9.945472 | 1 |
| TCGCTAG | 4205 | 0.0 | 9.791691 | 9 |
| GCCCGAT | 4140 | 0.0 | 9.773998 | 1 |
| CCCGGCT | 3030 | 0.0 | 9.605921 | 1 |
| GCGGGCT | 1950 | 0.0 | 9.465345 | 1 |
| CGCGCAT | 1880 | 0.0 | 9.440172 | 1 |
| CGCGGTA | 1855 | 1.8189894E-12 | 9.184703 | 1 |
| CGCGGGC | 775 | 3.9750384E-4 | 9.160012 | 1 |
| CGCGGAT | 2025 | 0.0 | 9.114778 | 1 |

Produced by FastQC (version 0.11.5)
