# Supplementary material for: Pitfall Flower Development and Organ Identity of Ceropegia sandersonii (Apocynaceae-Asclepiadoideae)
Source: Plants (Basel). 2020 Dec 14;9(12):1767. doi: 10.3390/plants9121767 (PMC7764971; doi:10.3390/plants9121767)
Supplement: Supplementary file 1 [file plants-09-01767-s001.zip › plants-1003419_Revision1_Supplementary Material/Folder S1_FastQC.zip!/FastQC/CerA21_reverse_paired_fastqc.html]

CerA21\_reverse\_paired.fq FastQC Report 

FastQC Report

Wed 13 Feb 2019  
CerA21\_reverse\_paired.fq

## Summary

- Basic Statistics
- Per base sequence quality
- Per tile sequence quality
- Per sequence quality scores
- Per base sequence content
- Per sequence GC content
- Per base N content
- Sequence Length Distribution
- Sequence Duplication Levels
- Overrepresented sequences
- Adapter Content
- Kmer Content

## Basic Statistics

| Measure | Value |
| --- | --- |
| Filename | CerA21\_reverse\_paired.fq |
| File type | Conventional base calls |
| Encoding | Sanger / Illumina 1.9 |
| Total Sequences | 21167042 |
| Sequences flagged as poor quality | 0 |
| Sequence length | 50-150 |
| %GC | 41 |

## Per base sequence quality

## Per tile sequence quality

## Per sequence quality scores

## Per base sequence content

## Per sequence GC content

## Per base N content

## Sequence Length Distribution

## Sequence Duplication Levels

## Overrepresented sequences

No overrepresented sequences

## Adapter Content

## Kmer Content

| Sequence | Count | PValue | Obs/Exp Max | Max Obs/Exp Position |
| --- | --- | --- | --- | --- |
| CGCGGGG | 555 | 0.0 | 25.091652 | 1 |
| AGATCGG | 14230 | 0.0 | 12.651171 | 140-144 |
| CGGGGGG | 1770 | 0.0 | 11.801582 | 1 |
| CGGGCCT | 2915 | 0.0 | 11.704415 | 1 |
| CGGGGAT | 5930 | 0.0 | 11.624458 | 1 |
| CGCACAT | 3565 | 0.0 | 11.523509 | 1 |
| CTCGCTA | 5070 | 0.0 | 11.258782 | 9 |
| CGCGTTT | 2120 | 0.0 | 11.166968 | 1 |
| CGCGGAT | 2265 | 0.0 | 11.066914 | 1 |
| CGGGCAT | 5815 | 0.0 | 11.016163 | 1 |
| CGCCCTT | 5185 | 0.0 | 10.608905 | 1 |
| GGAAAAT | 48160 | 0.0 | 10.597654 | 1 |
| CCCGGCT | 2715 | 0.0 | 10.514927 | 1 |
| CGCCCAT | 5860 | 0.0 | 10.456282 | 1 |
| CCCGAAT | 7435 | 0.0 | 10.395233 | 1 |
| CCCGACT | 4115 | 0.0 | 9.983306 | 1 |
| CCCGGTT | 5770 | 0.0 | 9.65398 | 1 |
| CCGGGTT | 5785 | 0.0 | 9.628948 | 1 |
| CGCCCCT | 3625 | 0.0 | 9.604046 | 1 |
| CTCGGAT | 8490 | 0.0 | 9.513548 | 1 |

Produced by FastQC (version 0.11.5)
