# Supplementary material for: Pitfall Flower Development and Organ Identity of Ceropegia sandersonii (Apocynaceae-Asclepiadoideae)
Source: Plants (Basel). 2020 Dec 14;9(12):1767. doi: 10.3390/plants9121767 (PMC7764971; doi:10.3390/plants9121767)
Supplement: Supplementary file 1 [file plants-09-01767-s001.zip › plants-1003419_Revision1_Supplementary Material/Folder S1_FastQC.zip!/FastQC/CerA58_forward_paired_fastqc.html]

CerA58\_forward\_paired.fq FastQC Report 

FastQC Report

Wed 13 Feb 2019  
CerA58\_forward\_paired.fq

## Summary

- Basic Statistics
- Per base sequence quality
- Per tile sequence quality
- Per sequence quality scores
- Per base sequence content
- Per sequence GC content
- Per base N content
- Sequence Length Distribution
- Sequence Duplication Levels
- Overrepresented sequences
- Adapter Content
- Kmer Content

## Basic Statistics

| Measure | Value |
| --- | --- |
| Filename | CerA58\_forward\_paired.fq |
| File type | Conventional base calls |
| Encoding | Sanger / Illumina 1.9 |
| Total Sequences | 22885141 |
| Sequences flagged as poor quality | 0 |
| Sequence length | 50-150 |
| %GC | 41 |

## Per base sequence quality

## Per tile sequence quality

## Per sequence quality scores

## Per base sequence content

## Per sequence GC content

## Per base N content

## Sequence Length Distribution

## Sequence Duplication Levels

## Overrepresented sequences

No overrepresented sequences

## Adapter Content

## Kmer Content

| Sequence | Count | PValue | Obs/Exp Max | Max Obs/Exp Position |
| --- | --- | --- | --- | --- |
| CTCGCTA | 7110 | 0.0 | 17.169172 | 9 |
| CGCGGGG | 610 | 1.0124495E-8 | 15.125765 | 1 |
| TCTCGCT | 10285 | 0.0 | 12.145039 | 8 |
| CGGGCAT | 6385 | 0.0 | 11.893967 | 1 |
| AGATCGG | 16780 | 0.0 | 11.630449 | 140-144 |
| CCCGCCT | 3750 | 0.0 | 11.355959 | 1 |
| TCGCTAG | 5180 | 0.0 | 11.235041 | 9 |
| CGCGAGT | 1650 | 0.0 | 11.183899 | 1 |
| GCCCGAT | 4075 | 0.0 | 10.972782 | 1 |
| CGCGTCC | 1300 | 1.8189894E-11 | 10.91919 | 1 |
| GGAAAAT | 54805 | 0.0 | 10.839497 | 1 |
| TTCTCGC | 12310 | 0.0 | 10.608735 | 7 |
| CGGGGAT | 5925 | 0.0 | 10.182032 | 1 |
| CGGGAAT | 12790 | 0.0 | 9.988627 | 1 |
| CGCGGTT | 2655 | 0.0 | 9.891019 | 1 |
| CCCGTAT | 4040 | 0.0 | 9.838083 | 1 |
| CGCGTTT | 2115 | 0.0 | 9.731762 | 1 |
| CCCGTCT | 4745 | 0.0 | 9.722568 | 1 |
| CGCCCTT | 5780 | 0.0 | 9.7007 | 1 |
| CGGGCCA | 2505 | 0.0 | 9.633299 | 1 |

Produced by FastQC (version 0.11.5)
