# Supplementary material for: Pitfall Flower Development and Organ Identity of Ceropegia sandersonii (Apocynaceae-Asclepiadoideae)
Source: Plants (Basel). 2020 Dec 14;9(12):1767. doi: 10.3390/plants9121767 (PMC7764971; doi:10.3390/plants9121767)
Supplement: Supplementary file 1 [file plants-09-01767-s001.zip › plants-1003419_Revision1_Supplementary Material/Folder S1_FastQC.zip!/FastQC/CerA58_reverse_paired_fastqc.html]

CerA58\_reverse\_paired.fq FastQC Report 

FastQC Report

Wed 13 Feb 2019  
CerA58\_reverse\_paired.fq

## Summary

- Basic Statistics
- Per base sequence quality
- Per tile sequence quality
- Per sequence quality scores
- Per base sequence content
- Per sequence GC content
- Per base N content
- Sequence Length Distribution
- Sequence Duplication Levels
- Overrepresented sequences
- Adapter Content
- Kmer Content

## Basic Statistics

| Measure | Value |
| --- | --- |
| Filename | CerA58\_reverse\_paired.fq |
| File type | Conventional base calls |
| Encoding | Sanger / Illumina 1.9 |
| Total Sequences | 22885141 |
| Sequences flagged as poor quality | 0 |
| Sequence length | 50-150 |
| %GC | 41 |

## Per base sequence quality

## Per tile sequence quality

## Per sequence quality scores

## Per base sequence content

## Per sequence GC content

## Per base N content

## Sequence Length Distribution

## Sequence Duplication Levels

## Overrepresented sequences

No overrepresented sequences

## Adapter Content

## Kmer Content

| Sequence | Count | PValue | Obs/Exp Max | Max Obs/Exp Position |
| --- | --- | --- | --- | --- |
| CGCGGGG | 710 | 0.0 | 22.523191 | 1 |
| CTCGCTA | 6765 | 0.0 | 16.542995 | 9 |
| CGCGTTT | 2335 | 0.0 | 15.186011 | 1 |
| AGATCGG | 16100 | 0.0 | 13.125861 | 140-144 |
| CGGGCAT | 6140 | 0.0 | 12.229701 | 1 |
| CGCGTAT | 1565 | 0.0 | 11.995265 | 1 |
| CGGGAAT | 12495 | 0.0 | 11.407172 | 1 |
| GGAAAAT | 52220 | 0.0 | 11.34392 | 1 |
| TCGCTAG | 4995 | 0.0 | 11.272119 | 9 |
| CGCGTCT | 1365 | 0.0 | 11.205996 | 1 |
| TCTCGCT | 10485 | 0.0 | 11.005143 | 8 |
| CGCGGGA | 1270 | 1.8189894E-11 | 10.949308 | 1 |
| CGCGAAT | 3005 | 0.0 | 10.874613 | 1 |
| CGCCAAT | 9715 | 0.0 | 10.806737 | 1 |
| CGCCCTT | 5945 | 0.0 | 10.759607 | 1 |
| CGGGGAT | 5900 | 0.0 | 10.488138 | 1 |
| GCCCGAT | 3920 | 0.0 | 10.46469 | 1 |
| CAGATCG | 15525 | 0.0 | 10.305192 | 140-144 |
| CGCCCAT | 6580 | 0.0 | 10.249585 | 1 |
| CGGGGAC | 5775 | 0.0 | 10.233575 | 1 |

Produced by FastQC (version 0.11.5)
