# Supplementary material for: Pitfall Flower Development and Organ Identity of Ceropegia sandersonii (Apocynaceae-Asclepiadoideae)
Source: Plants (Basel). 2020 Dec 14;9(12):1767. doi: 10.3390/plants9121767 (PMC7764971; doi:10.3390/plants9121767)
Supplement: Supplementary file 1 [file plants-09-01767-s001.zip › plants-1003419_Revision1_Supplementary Material/Folder S1_FastQC.zip!/FastQC/CerA78_forward_paired_fastqc.html]

CerA78\_forward\_paired.fq FastQC Report 

FastQC Report

Wed 13 Feb 2019  
CerA78\_forward\_paired.fq

## Summary

- Basic Statistics
- Per base sequence quality
- Per tile sequence quality
- Per sequence quality scores
- Per base sequence content
- Per sequence GC content
- Per base N content
- Sequence Length Distribution
- Sequence Duplication Levels
- Overrepresented sequences
- Adapter Content
- Kmer Content

## Basic Statistics

| Measure | Value |
| --- | --- |
| Filename | CerA78\_forward\_paired.fq |
| File type | Conventional base calls |
| Encoding | Sanger / Illumina 1.9 |
| Total Sequences | 21951933 |
| Sequences flagged as poor quality | 0 |
| Sequence length | 50-150 |
| %GC | 41 |

## Per base sequence quality

## Per tile sequence quality

## Per sequence quality scores

## Per base sequence content

## Per sequence GC content

## Per base N content

## Sequence Length Distribution

## Sequence Duplication Levels

## Overrepresented sequences

No overrepresented sequences

## Adapter Content

## Kmer Content

| Sequence | Count | PValue | Obs/Exp Max | Max Obs/Exp Position |
| --- | --- | --- | --- | --- |
| CGCGGGG | 580 | 0.0 | 23.241589 | 1 |
| CTCGCTA | 5765 | 0.0 | 13.660198 | 9 |
| CGGGCAT | 6110 | 0.0 | 12.7729645 | 1 |
| CGCCCTT | 5625 | 0.0 | 11.477811 | 1 |
| TCGCTAG | 4640 | 0.0 | 11.467706 | 9 |
| CCCGCTT | 3815 | 0.0 | 10.972301 | 1 |
| GGAAAAT | 52200 | 0.0 | 10.356778 | 1 |
| CGGGTAT | 4560 | 0.0 | 10.26879 | 1 |
| CGGGGGG | 2080 | 0.0 | 10.232885 | 1 |
| CGCACAT | 3615 | 0.0 | 10.2055235 | 1 |
| AGATCGG | 14200 | 0.0 | 10.073301 | 140-144 |
| CGGGGAT | 6015 | 0.0 | 9.79 | 1 |
| GGGAAAT | 29935 | 0.0 | 9.764684 | 1 |
| CCGGGAT | 7240 | 0.0 | 9.701454 | 1 |
| CGGGCAC | 3390 | 0.0 | 9.627164 | 1 |
| CCCGCAT | 3340 | 0.0 | 9.558864 | 1 |
| CGGGCCC | 1195 | 6.659502E-8 | 9.499315 | 1 |
| CGCGGGA | 1345 | 4.2346073E-9 | 9.494901 | 1 |
| GCCCGAT | 4035 | 0.0 | 9.494901 | 1 |
| CGCCCAT | 5940 | 0.0 | 9.435846 | 1 |

Produced by FastQC (version 0.11.5)
