# Supplementary material for: Pitfall Flower Development and Organ Identity of Ceropegia sandersonii (Apocynaceae-Asclepiadoideae)
Source: Plants (Basel). 2020 Dec 14;9(12):1767. doi: 10.3390/plants9121767 (PMC7764971; doi:10.3390/plants9121767)
Supplement: Supplementary file 1 [file plants-09-01767-s001.zip › plants-1003419_Revision1_Supplementary Material/Folder S1_FastQC.zip!/FastQC/CerA78_reverse_paired_fastqc.html]

CerA78\_reverse\_paired.fq FastQC Report 

FastQC Report

Wed 13 Feb 2019  
CerA78\_reverse\_paired.fq

## Summary

- Basic Statistics
- Per base sequence quality
- Per tile sequence quality
- Per sequence quality scores
- Per base sequence content
- Per sequence GC content
- Per base N content
- Sequence Length Distribution
- Sequence Duplication Levels
- Overrepresented sequences
- Adapter Content
- Kmer Content

## Basic Statistics

| Measure | Value |
| --- | --- |
| Filename | CerA78\_reverse\_paired.fq |
| File type | Conventional base calls |
| Encoding | Sanger / Illumina 1.9 |
| Total Sequences | 21951933 |
| Sequences flagged as poor quality | 0 |
| Sequence length | 50-150 |
| %GC | 41 |

## Per base sequence quality

## Per tile sequence quality

## Per sequence quality scores

## Per base sequence content

## Per sequence GC content

## Per base N content

## Sequence Length Distribution

## Sequence Duplication Levels

## Overrepresented sequences

No overrepresented sequences

## Adapter Content

## Kmer Content

| Sequence | Count | PValue | Obs/Exp Max | Max Obs/Exp Position |
| --- | --- | --- | --- | --- |
| CGCGGGG | 605 | 0.0 | 23.071049 | 1 |
| CGGGCCC | 1170 | 0.0 | 14.912376 | 1 |
| CTCGCTA | 5695 | 0.0 | 14.333795 | 9 |
| CCCGGCT | 3070 | 0.0 | 12.503081 | 1 |
| CGGGCAT | 5860 | 0.0 | 12.385925 | 1 |
| CGGGGGG | 1900 | 0.0 | 12.121408 | 1 |
| CGGGGAT | 5780 | 0.0 | 11.953638 | 1 |
| AGATCGG | 13480 | 0.0 | 11.234625 | 140-144 |
| CGCCCGT | 1430 | 0.0 | 11.224954 | 1 |
| CGCGTTT | 2390 | 0.0 | 11.096307 | 1 |
| CGCCCTT | 5905 | 0.0 | 10.991469 | 1 |
| CCGGGAT | 7055 | 0.0 | 10.782569 | 1 |
| CGCGTCT | 1485 | 3.6379788E-12 | 10.339248 | 1 |
| GCCCGAT | 4075 | 0.0 | 10.275817 | 1 |
| GGAAAAT | 50295 | 0.0 | 10.226697 | 1 |
| TCGCTAG | 4435 | 0.0 | 10.2256 | 9 |
| CGGGCAC | 3275 | 0.0 | 10.015653 | 1 |
| CGCACAT | 3520 | 0.0 | 9.9133415 | 1 |
| CGCCCCT | 3870 | 0.0 | 9.738129 | 1 |
| CGGGTAT | 4250 | 0.0 | 9.688483 | 1 |

Produced by FastQC (version 0.11.5)
