# Supplementary material for: Pitfall Flower Development and Organ Identity of Ceropegia sandersonii (Apocynaceae-Asclepiadoideae)
Source: Plants (Basel). 2020 Dec 14;9(12):1767. doi: 10.3390/plants9121767 (PMC7764971; doi:10.3390/plants9121767)
Supplement: Supplementary file 1 [file plants-09-01767-s001.zip › plants-1003419_Revision1_Supplementary Material/Folder S1_FastQC.zip!/FastQC/CerB72_forward_paired_fastqc.html]

CerB72\_forward\_paired.fq FastQC Report 

FastQC Report

Wed 13 Feb 2019  
CerB72\_forward\_paired.fq

## Summary

- Basic Statistics
- Per base sequence quality
- Per tile sequence quality
- Per sequence quality scores
- Per base sequence content
- Per sequence GC content
- Per base N content
- Sequence Length Distribution
- Sequence Duplication Levels
- Overrepresented sequences
- Adapter Content
- Kmer Content

## Basic Statistics

| Measure | Value |
| --- | --- |
| Filename | CerB72\_forward\_paired.fq |
| File type | Conventional base calls |
| Encoding | Sanger / Illumina 1.9 |
| Total Sequences | 19008814 |
| Sequences flagged as poor quality | 0 |
| Sequence length | 50-150 |
| %GC | 41 |

## Per base sequence quality

## Per tile sequence quality

## Per sequence quality scores

## Per base sequence content

## Per sequence GC content

## Per base N content

## Sequence Length Distribution

## Sequence Duplication Levels

## Overrepresented sequences

No overrepresented sequences

## Adapter Content

## Kmer Content

| Sequence | Count | PValue | Obs/Exp Max | Max Obs/Exp Position |
| --- | --- | --- | --- | --- |
| CGGGCAT | 5225 | 0.0 | 16.29057 | 1 |
| CGCGGGG | 540 | 6.4285086E-7 | 14.44908 | 1 |
| CGCCCTT | 4885 | 0.0 | 12.923101 | 1 |
| AATCCGC | 5250 | 0.0 | 11.88953 | 5 |
| CGCACAT | 2985 | 0.0 | 11.881382 | 1 |
| CGCGGTT | 1735 | 0.0 | 11.856044 | 1 |
| CGCCCAT | 4690 | 0.0 | 11.645528 | 1 |
| CGGGGAT | 4420 | 0.0 | 10.912593 | 1 |
| CGCCCCT | 2955 | 0.0 | 10.8018055 | 1 |
| CGGGAAT | 8890 | 0.0 | 10.771428 | 1 |
| CGCCTAT | 2705 | 0.0 | 10.751224 | 1 |
| CGGGCTT | 4225 | 0.0 | 10.744706 | 1 |
| CCCGGCT | 2650 | 0.0 | 10.706695 | 1 |
| CGCGTTT | 2005 | 0.0 | 10.613244 | 1 |
| CCCGGTT | 5030 | 0.0 | 10.57632 | 1 |
| CCCGCAT | 2470 | 0.0 | 10.338245 | 1 |
| TCCGCTC | 4875 | 0.0 | 10.330588 | 7 |
| AGATCGG | 10010 | 0.0 | 10.30868 | 140-144 |
| TGCGCGC | 985 | 4.7103276E-7 | 10.081367 | 9 |
| CCGGGAT | 5800 | 0.0 | 9.906 | 1 |

Produced by FastQC (version 0.11.5)
