# Supplementary material for: Pitfall Flower Development and Organ Identity of Ceropegia sandersonii (Apocynaceae-Asclepiadoideae)
Source: Plants (Basel). 2020 Dec 14;9(12):1767. doi: 10.3390/plants9121767 (PMC7764971; doi:10.3390/plants9121767)
Supplement: Supplementary file 1 [file plants-09-01767-s001.zip › plants-1003419_Revision1_Supplementary Material/Folder S1_FastQC.zip!/FastQC/CerB72_reverse_paired_fastqc.html]

CerB72\_reverse\_paired.fq FastQC Report 

FastQC Report

Wed 13 Feb 2019  
CerB72\_reverse\_paired.fq

## Summary

- Basic Statistics
- Per base sequence quality
- Per tile sequence quality
- Per sequence quality scores
- Per base sequence content
- Per sequence GC content
- Per base N content
- Sequence Length Distribution
- Sequence Duplication Levels
- Overrepresented sequences
- Adapter Content
- Kmer Content

## Basic Statistics

| Measure | Value |
| --- | --- |
| Filename | CerB72\_reverse\_paired.fq |
| File type | Conventional base calls |
| Encoding | Sanger / Illumina 1.9 |
| Total Sequences | 19008814 |
| Sequences flagged as poor quality | 0 |
| Sequence length | 50-150 |
| %GC | 41 |

## Per base sequence quality

## Per tile sequence quality

## Per sequence quality scores

## Per base sequence content

## Per sequence GC content

## Per base N content

## Sequence Length Distribution

## Sequence Duplication Levels

## Overrepresented sequences

No overrepresented sequences

## Adapter Content

## Kmer Content

| Sequence | Count | PValue | Obs/Exp Max | Max Obs/Exp Position |
| --- | --- | --- | --- | --- |
| CGCGGGG | 555 | 0.0 | 20.074203 | 1 |
| CGGGCAT | 4935 | 0.0 | 15.661997 | 1 |
| CCCGGCT | 2460 | 0.0 | 14.719042 | 1 |
| TCCGCTC | 4770 | 0.0 | 14.011596 | 7 |
| CGCGTAT | 1125 | 0.0 | 13.617001 | 1 |
| AATCCGC | 5300 | 0.0 | 13.135769 | 5 |
| CGCGTTT | 1960 | 0.0 | 12.434356 | 1 |
| AGATCGG | 9380 | 0.0 | 12.060377 | 140-144 |
| CGCGCAC | 930 | 1.7898856E-9 | 11.979767 | 1 |
| CGCGCAT | 1230 | 9.094947E-12 | 11.32234 | 1 |
| CGCCCTT | 4945 | 0.0 | 11.265099 | 1 |
| CGGGAAT | 8280 | 0.0 | 10.932623 | 1 |
| CGCTCTC | 6140 | 0.0 | 10.885231 | 9 |
| CCGCTCT | 6075 | 0.0 | 10.657895 | 8 |
| ATCCGCT | 7525 | 0.0 | 10.362064 | 6 |
| GCCCGAT | 3970 | 0.0 | 10.348391 | 1 |
| CCCGCAT | 2780 | 0.0 | 10.269526 | 1 |
| CGCCCAT | 4890 | 0.0 | 10.252623 | 1 |
| CAATCCG | 8030 | 0.0 | 9.883725 | 4 |
| CGCACAC | 2690 | 0.0 | 9.836546 | 1 |

Produced by FastQC (version 0.11.5)
