# Supplementary material for: Pitfall Flower Development and Organ Identity of Ceropegia sandersonii (Apocynaceae-Asclepiadoideae)
Source: Plants (Basel). 2020 Dec 14;9(12):1767. doi: 10.3390/plants9121767 (PMC7764971; doi:10.3390/plants9121767)
Supplement: Supplementary file 1 [file plants-09-01767-s001.zip › plants-1003419_Revision1_Supplementary Material/Folder S1_FastQC.zip!/FastQC/CerB79_forward_paired_fastqc.html]

CerB79\_forward\_paired.fq FastQC Report 

FastQC Report

Wed 13 Feb 2019  
CerB79\_forward\_paired.fq

## Summary

- Basic Statistics
- Per base sequence quality
- Per tile sequence quality
- Per sequence quality scores
- Per base sequence content
- Per sequence GC content
- Per base N content
- Sequence Length Distribution
- Sequence Duplication Levels
- Overrepresented sequences
- Adapter Content
- Kmer Content

## Basic Statistics

| Measure | Value |
| --- | --- |
| Filename | CerB79\_forward\_paired.fq |
| File type | Conventional base calls |
| Encoding | Sanger / Illumina 1.9 |
| Total Sequences | 18598712 |
| Sequences flagged as poor quality | 0 |
| Sequence length | 50-150 |
| %GC | 41 |

## Per base sequence quality

## Per tile sequence quality

## Per sequence quality scores

## Per base sequence content

## Per sequence GC content

## Per base N content

## Sequence Length Distribution

## Sequence Duplication Levels

## Overrepresented sequences

No overrepresented sequences

## Adapter Content

## Kmer Content

| Sequence | Count | PValue | Obs/Exp Max | Max Obs/Exp Position |
| --- | --- | --- | --- | --- |
| CGGGCAT | 5435 | 0.0 | 22.568335 | 1 |
| TCCGCTC | 5405 | 0.0 | 18.36476 | 7 |
| CGCGGGG | 545 | 1.1823431E-10 | 18.213125 | 1 |
| AATCCGC | 5715 | 0.0 | 17.864843 | 5 |
| CCGCTCT | 6340 | 0.0 | 14.761503 | 8 |
| CGCTCTC | 6685 | 0.0 | 14.4239235 | 9 |
| GCCCGGG | 2385 | 0.0 | 14.2694025 | 5 |
| CGGGGAT | 4635 | 0.0 | 12.849389 | 1 |
| CGGGGGG | 1715 | 0.0 | 12.815942 | 1 |
| ATCCGCT | 9035 | 0.0 | 12.712759 | 6 |
| AGATCGG | 10980 | 0.0 | 12.634328 | 140-144 |
| CAATCCG | 8010 | 0.0 | 12.480718 | 4 |
| CCCGGGC | 2650 | 0.0 | 12.30736 | 6 |
| CGCGCAC | 825 | 4.0938176E-8 | 12.031701 | 1 |
| CCCGCAT | 2785 | 0.0 | 11.456191 | 1 |
| CCGGGAT | 5710 | 0.0 | 11.175303 | 1 |
| CGCGTAT | 1275 | 1.2732926E-11 | 11.12174 | 1 |
| CGGGCTT | 3990 | 0.0 | 11.017213 | 1 |
| CGCGAAT | 2670 | 0.0 | 10.887434 | 1 |
| CCCGCCT | 2810 | 0.0 | 10.849634 | 1 |

Produced by FastQC (version 0.11.5)
