# Supplementary material for: Pitfall Flower Development and Organ Identity of Ceropegia sandersonii (Apocynaceae-Asclepiadoideae)
Source: Plants (Basel). 2020 Dec 14;9(12):1767. doi: 10.3390/plants9121767 (PMC7764971; doi:10.3390/plants9121767)
Supplement: Supplementary file 1 [file plants-09-01767-s001.zip › plants-1003419_Revision1_Supplementary Material/Folder S1_FastQC.zip!/FastQC/CerB79_reverse_paired_fastqc.html]

CerB79\_reverse\_paired.fq FastQC Report 

FastQC Report

Wed 13 Feb 2019  
CerB79\_reverse\_paired.fq

## Summary

- Basic Statistics
- Per base sequence quality
- Per tile sequence quality
- Per sequence quality scores
- Per base sequence content
- Per sequence GC content
- Per base N content
- Sequence Length Distribution
- Sequence Duplication Levels
- Overrepresented sequences
- Adapter Content
- Kmer Content

## Basic Statistics

| Measure | Value |
| --- | --- |
| Filename | CerB79\_reverse\_paired.fq |
| File type | Conventional base calls |
| Encoding | Sanger / Illumina 1.9 |
| Total Sequences | 18598712 |
| Sequences flagged as poor quality | 0 |
| Sequence length | 50-150 |
| %GC | 41 |

## Per base sequence quality

## Per tile sequence quality

## Per sequence quality scores

## Per base sequence content

## Per sequence GC content

## Per base N content

## Sequence Length Distribution

## Sequence Duplication Levels

## Overrepresented sequences

No overrepresented sequences

## Adapter Content

## Kmer Content

| Sequence | Count | PValue | Obs/Exp Max | Max Obs/Exp Position |
| --- | --- | --- | --- | --- |
| CGCGGGG | 705 | 0.0 | 25.719158 | 1 |
| CGGGCAT | 5195 | 0.0 | 25.505896 | 1 |
| AATCCGC | 5615 | 0.0 | 21.23215 | 5 |
| TCCGCTC | 5675 | 0.0 | 19.902216 | 7 |
| CCGCTCT | 6435 | 0.0 | 16.793276 | 8 |
| CGCTCTC | 6660 | 0.0 | 16.330618 | 9 |
| CAATCCG | 8300 | 0.0 | 15.371654 | 4 |
| ATCCGCT | 8790 | 0.0 | 15.070128 | 6 |
| CGGGGGG | 1725 | 0.0 | 14.554119 | 1 |
| CGGGGAT | 4965 | 0.0 | 13.7651 | 1 |
| CGCGTAT | 1270 | 0.0 | 13.728048 | 1 |
| CGCGGAT | 1970 | 0.0 | 13.452093 | 1 |
| AGATCGG | 10930 | 0.0 | 13.33275 | 140-144 |
| CGCGGCT | 1565 | 0.0 | 12.922787 | 1 |
| CGGGAAT | 8755 | 0.0 | 12.107654 | 1 |
| CGCCCTT | 4915 | 0.0 | 11.918683 | 1 |
| CCGGGAT | 5715 | 0.0 | 11.714602 | 1 |
| CGGGGAC | 3115 | 0.0 | 11.193979 | 1 |
| CGGGCCC | 1010 | 6.381015E-9 | 11.047681 | 1 |
| CGCGGTT | 1800 | 0.0 | 10.848209 | 1 |

Produced by FastQC (version 0.11.5)
