# Supplementary material for: Pitfall Flower Development and Organ Identity of Ceropegia sandersonii (Apocynaceae-Asclepiadoideae)
Source: Plants (Basel). 2020 Dec 14;9(12):1767. doi: 10.3390/plants9121767 (PMC7764971; doi:10.3390/plants9121767)
Supplement: Supplementary file 1 [file plants-09-01767-s001.zip › plants-1003419_Revision1_Supplementary Material/Folder S1_FastQC.zip!/FastQC/CerB81_forward_paired_fastqc.html]

CerB81\_forward\_paired.fq FastQC Report 

FastQC Report

Wed 13 Feb 2019  
CerB81\_forward\_paired.fq

## Summary

- Basic Statistics
- Per base sequence quality
- Per tile sequence quality
- Per sequence quality scores
- Per base sequence content
- Per sequence GC content
- Per base N content
- Sequence Length Distribution
- Sequence Duplication Levels
- Overrepresented sequences
- Adapter Content
- Kmer Content

## Basic Statistics

| Measure | Value |
| --- | --- |
| Filename | CerB81\_forward\_paired.fq |
| File type | Conventional base calls |
| Encoding | Sanger / Illumina 1.9 |
| Total Sequences | 21618075 |
| Sequences flagged as poor quality | 0 |
| Sequence length | 50-150 |
| %GC | 41 |

## Per base sequence quality

## Per tile sequence quality

## Per sequence quality scores

## Per base sequence content

## Per sequence GC content

## Per base N content

## Sequence Length Distribution

## Sequence Duplication Levels

## Overrepresented sequences

No overrepresented sequences

## Adapter Content

## Kmer Content

| Sequence | Count | PValue | Obs/Exp Max | Max Obs/Exp Position |
| --- | --- | --- | --- | --- |
| CGCGGGG | 575 | 0.0 | 19.75013 | 1 |
| CGGGCAT | 5580 | 0.0 | 16.917465 | 1 |
| CGCGTAT | 1375 | 0.0 | 14.453506 | 1 |
| CCCGCAT | 2860 | 0.0 | 12.904916 | 1 |
| CGCCCTT | 5485 | 0.0 | 12.681402 | 1 |
| CGGGGAT | 5245 | 0.0 | 12.179091 | 1 |
| AATCCGC | 6305 | 0.0 | 12.157843 | 5 |
| CGGGCCC | 1015 | 4.1472958E-10 | 11.887779 | 1 |
| TCCGCTC | 5670 | 0.0 | 11.766915 | 7 |
| CGGGAAT | 9715 | 0.0 | 11.762535 | 1 |
| CCCGTAT | 3445 | 0.0 | 10.919544 | 1 |
| AGATCGG | 11545 | 0.0 | 10.732051 | 140-144 |
| CGGGCAC | 3025 | 0.0 | 10.558567 | 1 |
| CGCCCAT | 5675 | 0.0 | 10.505852 | 1 |
| CGCCCGT | 1190 | 5.7480065E-9 | 10.139577 | 1 |
| CGGGCCT | 2865 | 0.0 | 9.909534 | 1 |
| CGCGTTT | 2295 | 0.0 | 9.89658 | 1 |
| CGCACAT | 3470 | 0.0 | 9.818149 | 1 |
| CCGCTCT | 6760 | 0.0 | 9.44943 | 8 |
| CGCCCCT | 3185 | 0.0 | 9.359609 | 1 |

Produced by FastQC (version 0.11.5)
