# Supplementary material for: Pitfall Flower Development and Organ Identity of Ceropegia sandersonii (Apocynaceae-Asclepiadoideae)
Source: Plants (Basel). 2020 Dec 14;9(12):1767. doi: 10.3390/plants9121767 (PMC7764971; doi:10.3390/plants9121767)
Supplement: Supplementary file 1 [file plants-09-01767-s001.zip › plants-1003419_Revision1_Supplementary Material/Folder S1_FastQC.zip!/FastQC/CerB81_reverse_paired_fastqc.html]

CerB81\_reverse\_paired.fq FastQC Report 

FastQC Report

Wed 13 Feb 2019  
CerB81\_reverse\_paired.fq

## Summary

- Basic Statistics
- Per base sequence quality
- Per tile sequence quality
- Per sequence quality scores
- Per base sequence content
- Per sequence GC content
- Per base N content
- Sequence Length Distribution
- Sequence Duplication Levels
- Overrepresented sequences
- Adapter Content
- Kmer Content

## Basic Statistics

| Measure | Value |
| --- | --- |
| Filename | CerB81\_reverse\_paired.fq |
| File type | Conventional base calls |
| Encoding | Sanger / Illumina 1.9 |
| Total Sequences | 21618075 |
| Sequences flagged as poor quality | 0 |
| Sequence length | 50-150 |
| %GC | 41 |

## Per base sequence quality

## Per tile sequence quality

## Per sequence quality scores

## Per base sequence content

## Per sequence GC content

## Per base N content

## Sequence Length Distribution

## Sequence Duplication Levels

## Overrepresented sequences

No overrepresented sequences

## Adapter Content

## Kmer Content

| Sequence | Count | PValue | Obs/Exp Max | Max Obs/Exp Position |
| --- | --- | --- | --- | --- |
| CGCGGGG | 710 | 0.0 | 24.538317 | 1 |
| CGGGCAT | 5605 | 0.0 | 16.90933 | 1 |
| CGGGGAT | 5250 | 0.0 | 13.539541 | 1 |
| AATCCGC | 5995 | 0.0 | 12.900587 | 5 |
| CCCGCAT | 3000 | 0.0 | 12.543986 | 1 |
| CGGGAAT | 9425 | 0.0 | 11.5347 | 1 |
| AGATCGG | 11475 | 0.0 | 11.486185 | 140-144 |
| CGCCTCT | 7230 | 0.0 | 11.277443 | 1 |
| CGGGGTA | 2345 | 0.0 | 10.995678 | 1 |
| CGCGGAT | 2370 | 0.0 | 10.879689 | 1 |
| CGGGTAT | 4100 | 0.0 | 10.878254 | 1 |
| CGAAAAC | 12605 | 0.0 | 10.780895 | 1 |
| CGGGCGT | 1115 | 2.6611815E-9 | 10.625201 | 1 |
| TCCGCTC | 5650 | 0.0 | 10.6053915 | 7 |
| CCCGGCT | 2850 | 0.0 | 10.514454 | 1 |
| CCCGGAT | 6570 | 0.0 | 10.501055 | 1 |
| CGGGCTT | 4535 | 0.0 | 10.449481 | 1 |
| CGGGCCC | 1025 | 9.440373E-8 | 10.198363 | 1 |
| CGGGGGG | 1915 | 0.0 | 10.189487 | 1 |
| CGCCCCT | 3365 | 0.0 | 10.147851 | 1 |

Produced by FastQC (version 0.11.5)
