# Supplementary material for: Pitfall Flower Development and Organ Identity of Ceropegia sandersonii (Apocynaceae-Asclepiadoideae)
Source: Plants (Basel). 2020 Dec 14;9(12):1767. doi: 10.3390/plants9121767 (PMC7764971; doi:10.3390/plants9121767)
Supplement: Supplementary file 1 [file plants-09-01767-s001.zip › plants-1003419_Revision1_Supplementary Material/Folder S1_FastQC.zip!/FastQC/CerC17_forward_paired_fastqc.html]

CerC17\_forward\_paired.fq FastQC Report 

FastQC Report

Wed 13 Feb 2019  
CerC17\_forward\_paired.fq

## Summary

- Basic Statistics
- Per base sequence quality
- Per tile sequence quality
- Per sequence quality scores
- Per base sequence content
- Per sequence GC content
- Per base N content
- Sequence Length Distribution
- Sequence Duplication Levels
- Overrepresented sequences
- Adapter Content
- Kmer Content

## Basic Statistics

| Measure | Value |
| --- | --- |
| Filename | CerC17\_forward\_paired.fq |
| File type | Conventional base calls |
| Encoding | Sanger / Illumina 1.9 |
| Total Sequences | 20411892 |
| Sequences flagged as poor quality | 0 |
| Sequence length | 50-150 |
| %GC | 41 |

## Per base sequence quality

## Per tile sequence quality

## Per sequence quality scores

## Per base sequence content

## Per sequence GC content

## Per base N content

## Sequence Length Distribution

## Sequence Duplication Levels

## Overrepresented sequences

No overrepresented sequences

## Adapter Content

## Kmer Content

| Sequence | Count | PValue | Obs/Exp Max | Max Obs/Exp Position |
| --- | --- | --- | --- | --- |
| CGCGGGG | 545 | 5.456968E-12 | 19.558983 | 1 |
| CTCGAAC | 11305 | 0.0 | 17.91537 | 1 |
| CCCGGCT | 2005 | 0.0 | 14.886289 | 1 |
| CGAACAT | 10785 | 0.0 | 14.496196 | 3 |
| CGGGGGG | 1540 | 0.0 | 14.305153 | 1 |
| CCCGGTT | 9525 | 0.0 | 14.250165 | 1 |
| CGCCTCT | 5595 | 0.0 | 13.844521 | 1 |
| CGCGGTT | 2110 | 0.0 | 13.808703 | 1 |
| CGCGCAT | 1170 | 0.0 | 13.362518 | 1 |
| GGTTAAC | 12995 | 0.0 | 13.015241 | 1 |
| CGGGCTT | 3875 | 0.0 | 12.654031 | 1 |
| CGGGTAT | 3135 | 0.0 | 12.240741 | 1 |
| CGCGTGT | 945 | 1.6716513E-9 | 12.032052 | 1 |
| CGGGGCG | 905 | 1.1330485E-8 | 11.778614 | 1 |
| TCGAACA | 12560 | 0.0 | 11.768611 | 2 |
| CGGGGGT | 1520 | 0.0 | 11.688208 | 1 |
| AGATCGG | 10925 | 0.0 | 11.670326 | 140-144 |
| CGGGGAT | 4525 | 0.0 | 11.307469 | 1 |
| CGCGTAT | 945 | 2.1471351E-8 | 11.280048 | 1 |
| CCCGGAT | 4790 | 0.0 | 11.275338 | 1 |

Produced by FastQC (version 0.11.5)
