# Supplementary material for: Pitfall Flower Development and Organ Identity of Ceropegia sandersonii (Apocynaceae-Asclepiadoideae)
Source: Plants (Basel). 2020 Dec 14;9(12):1767. doi: 10.3390/plants9121767 (PMC7764971; doi:10.3390/plants9121767)
Supplement: Supplementary file 1 [file plants-09-01767-s001.zip › plants-1003419_Revision1_Supplementary Material/Folder S1_FastQC.zip!/FastQC/CerC17_reverse_paired_fastqc.html]

CerC17\_reverse\_paired.fq FastQC Report 

FastQC Report

Wed 13 Feb 2019  
CerC17\_reverse\_paired.fq

## Summary

- Basic Statistics
- Per base sequence quality
- Per tile sequence quality
- Per sequence quality scores
- Per base sequence content
- Per sequence GC content
- Per base N content
- Sequence Length Distribution
- Sequence Duplication Levels
- Overrepresented sequences
- Adapter Content
- Kmer Content

## Basic Statistics

| Measure | Value |
| --- | --- |
| Filename | CerC17\_reverse\_paired.fq |
| File type | Conventional base calls |
| Encoding | Sanger / Illumina 1.9 |
| Total Sequences | 20411892 |
| Sequences flagged as poor quality | 0 |
| Sequence length | 50-150 |
| %GC | 41 |

## Per base sequence quality

## Per tile sequence quality

## Per sequence quality scores

## Per base sequence content

## Per sequence GC content

## Per base N content

## Sequence Length Distribution

## Sequence Duplication Levels

## Overrepresented sequences

No overrepresented sequences

## Adapter Content

## Kmer Content

| Sequence | Count | PValue | Obs/Exp Max | Max Obs/Exp Position |
| --- | --- | --- | --- | --- |
| CGCGGGG | 540 | 0.0 | 22.000631 | 1 |
| CGGGGAT | 4500 | 0.0 | 17.704039 | 1 |
| CTCGAAC | 10975 | 0.0 | 17.701914 | 1 |
| CCCGGTT | 9020 | 0.0 | 16.42515 | 1 |
| CGCGGTT | 2160 | 0.0 | 15.853396 | 1 |
| CGCCTCT | 5955 | 0.0 | 14.434552 | 1 |
| CGAACAT | 10800 | 0.0 | 13.714815 | 3 |
| AGATCGG | 10525 | 0.0 | 12.8597765 | 140-144 |
| CGGGCAC | 2490 | 0.0 | 12.6297035 | 1 |
| GGTTAAC | 13505 | 0.0 | 12.626275 | 1 |
| CGCGTAT | 1055 | 5.456968E-12 | 12.585809 | 1 |
| TCGAACA | 12035 | 0.0 | 12.426466 | 2 |
| CTCGAAT | 18400 | 0.0 | 12.229762 | 1 |
| CGGGGAG | 7850 | 0.0 | 12.107354 | 1 |
| CGGGCGT | 880 | 9.587893E-9 | 11.9121065 | 1 |
| CGCGCGT | 415 | 0.00371323 | 11.787724 | 1 |
| CGCCCTT | 7175 | 0.0 | 11.687977 | 1 |
| GATACGA | 10065 | 0.0 | 11.592677 | 7 |
| CGGGCTT | 4105 | 0.0 | 11.576459 | 1 |
| CGCTGAT | 4590 | 0.0 | 11.571266 | 1 |

Produced by FastQC (version 0.11.5)
