# Supplementary material for: Pitfall Flower Development and Organ Identity of Ceropegia sandersonii (Apocynaceae-Asclepiadoideae)
Source: Plants (Basel). 2020 Dec 14;9(12):1767. doi: 10.3390/plants9121767 (PMC7764971; doi:10.3390/plants9121767)
Supplement: Supplementary file 1 [file plants-09-01767-s001.zip › plants-1003419_Revision1_Supplementary Material/Folder S1_FastQC.zip!/FastQC/CerC25_forward_paired_fastqc.html]

CerC25\_forward\_paired.fq FastQC Report 

FastQC Report

Wed 13 Feb 2019  
CerC25\_forward\_paired.fq

## Summary

- Basic Statistics
- Per base sequence quality
- Per tile sequence quality
- Per sequence quality scores
- Per base sequence content
- Per sequence GC content
- Per base N content
- Sequence Length Distribution
- Sequence Duplication Levels
- Overrepresented sequences
- Adapter Content
- Kmer Content

## Basic Statistics

| Measure | Value |
| --- | --- |
| Filename | CerC25\_forward\_paired.fq |
| File type | Conventional base calls |
| Encoding | Sanger / Illumina 1.9 |
| Total Sequences | 19935070 |
| Sequences flagged as poor quality | 0 |
| Sequence length | 50-150 |
| %GC | 41 |

## Per base sequence quality

## Per tile sequence quality

## Per sequence quality scores

## Per base sequence content

## Per sequence GC content

## Per base N content

## Sequence Length Distribution

## Sequence Duplication Levels

## Overrepresented sequences

No overrepresented sequences

## Adapter Content

## Kmer Content

| Sequence | Count | PValue | Obs/Exp Max | Max Obs/Exp Position |
| --- | --- | --- | --- | --- |
| CGCGGGG | 595 | 0.0 | 35.82746 | 1 |
| CTCGAAC | 9850 | 0.0 | 23.734024 | 1 |
| CGAACAT | 10120 | 0.0 | 21.064566 | 3 |
| CGCCTCT | 5075 | 0.0 | 19.182106 | 1 |
| TCGAACA | 11450 | 0.0 | 18.245407 | 2 |
| CGCGGTT | 1710 | 0.0 | 17.868334 | 1 |
| CGGGGCG | 770 | 0.0 | 16.610914 | 1 |
| CGCGCCC | 705 | 1.6370905E-11 | 16.126593 | 1 |
| GGTTAAC | 14585 | 0.0 | 15.639049 | 1 |
| CGGGGAT | 3705 | 0.0 | 14.959536 | 1 |
| GCGGGTT | 2380 | 0.0 | 14.330984 | 1 |
| CCCGGCT | 1700 | 0.0 | 13.793572 | 1 |
| AGTCGCT | 9275 | 0.0 | 13.483744 | 6 |
| AGATCGG | 9225 | 0.0 | 13.348028 | 140-144 |
| CCCATAC | 12895 | 0.0 | 13.225016 | 8 |
| CGCGCAT | 1040 | 3.6379788E-12 | 12.981713 | 1 |
| CGCCCCT | 2190 | 0.0 | 12.978593 | 1 |
| GGCGCCC | 2585 | 0.0 | 12.644715 | 2 |
| GTCGCTT | 10085 | 0.0 | 12.330307 | 7 |
| CGCGTAT | 880 | 7.490598E-9 | 12.112124 | 1 |

Produced by FastQC (version 0.11.5)
