# Supplementary material for: Pitfall Flower Development and Organ Identity of Ceropegia sandersonii (Apocynaceae-Asclepiadoideae)
Source: Plants (Basel). 2020 Dec 14;9(12):1767. doi: 10.3390/plants9121767 (PMC7764971; doi:10.3390/plants9121767)
Supplement: Supplementary file 1 [file plants-09-01767-s001.zip › plants-1003419_Revision1_Supplementary Material/Folder S1_FastQC.zip!/FastQC/CerC25_reverse_paired_fastqc.html]

CerC25\_reverse\_paired.fq FastQC Report 

FastQC Report

Wed 13 Feb 2019  
CerC25\_reverse\_paired.fq

## Summary

- Basic Statistics
- Per base sequence quality
- Per tile sequence quality
- Per sequence quality scores
- Per base sequence content
- Per sequence GC content
- Per base N content
- Sequence Length Distribution
- Sequence Duplication Levels
- Overrepresented sequences
- Adapter Content
- Kmer Content

## Basic Statistics

| Measure | Value |
| --- | --- |
| Filename | CerC25\_reverse\_paired.fq |
| File type | Conventional base calls |
| Encoding | Sanger / Illumina 1.9 |
| Total Sequences | 19935070 |
| Sequences flagged as poor quality | 0 |
| Sequence length | 50-150 |
| %GC | 41 |

## Per base sequence quality

## Per tile sequence quality

## Per sequence quality scores

## Per base sequence content

## Per sequence GC content

## Per base N content

## Sequence Length Distribution

## Sequence Duplication Levels

## Overrepresented sequences

No overrepresented sequences

## Adapter Content

## Kmer Content

| Sequence | Count | PValue | Obs/Exp Max | Max Obs/Exp Position |
| --- | --- | --- | --- | --- |
| CTCGAAC | 10205 | 0.0 | 23.879063 | 1 |
| CGCCTCT | 5025 | 0.0 | 22.649385 | 1 |
| CGCGGGG | 515 | 0.0 | 21.6929 | 1 |
| CGAACAT | 10370 | 0.0 | 21.069801 | 3 |
| CGGGGGG | 1375 | 0.0 | 19.804632 | 1 |
| CGCGTAT | 845 | 0.0 | 19.005356 | 1 |
| CGCGCAT | 1080 | 0.0 | 18.749044 | 1 |
| TCGAACA | 11840 | 0.0 | 18.576492 | 2 |
| CGCGGTT | 1700 | 0.0 | 18.48283 | 1 |
| CGCGCCC | 680 | 0.0 | 18.48283 | 1 |
| CGCGCAG | 710 | 2.3646862E-11 | 15.734991 | 1 |
| CGGGGAT | 4020 | 0.0 | 15.632245 | 1 |
| CGGGCTT | 3400 | 0.0 | 15.402358 | 1 |
| CGCGCCT | 965 | 0.0 | 15.194866 | 1 |
| CCCATAC | 13390 | 0.0 | 15.066529 | 8 |
| CGCCCAT | 3770 | 0.0 | 14.81677 | 1 |
| GGCGCCC | 2650 | 0.0 | 14.491778 | 2 |
| GATACGA | 11520 | 0.0 | 14.421832 | 7 |
| CGGGGGT | 1325 | 0.0 | 14.2282915 | 1 |
| CCGGGTT | 3525 | 0.0 | 14.063846 | 1 |

Produced by FastQC (version 0.11.5)
