# Supplementary material for: Pitfall Flower Development and Organ Identity of Ceropegia sandersonii (Apocynaceae-Asclepiadoideae)
Source: Plants (Basel). 2020 Dec 14;9(12):1767. doi: 10.3390/plants9121767 (PMC7764971; doi:10.3390/plants9121767)
Supplement: Supplementary file 1 [file plants-09-01767-s001.zip › plants-1003419_Revision1_Supplementary Material/Folder S1_FastQC.zip!/FastQC/CerC38_forward_paired_fastqc.html]

CerC38\_forward\_paired.fq FastQC Report 

FastQC Report

Wed 13 Feb 2019  
CerC38\_forward\_paired.fq

## Summary

- Basic Statistics
- Per base sequence quality
- Per tile sequence quality
- Per sequence quality scores
- Per base sequence content
- Per sequence GC content
- Per base N content
- Sequence Length Distribution
- Sequence Duplication Levels
- Overrepresented sequences
- Adapter Content
- Kmer Content

## Basic Statistics

| Measure | Value |
| --- | --- |
| Filename | CerC38\_forward\_paired.fq |
| File type | Conventional base calls |
| Encoding | Sanger / Illumina 1.9 |
| Total Sequences | 17567580 |
| Sequences flagged as poor quality | 0 |
| Sequence length | 50-150 |
| %GC | 40 |

## Per base sequence quality

## Per tile sequence quality

## Per sequence quality scores

## Per base sequence content

## Per sequence GC content

## Per base N content

## Sequence Length Distribution

## Sequence Duplication Levels

## Overrepresented sequences

No overrepresented sequences

## Adapter Content

## Kmer Content

| Sequence | Count | PValue | Obs/Exp Max | Max Obs/Exp Position |
| --- | --- | --- | --- | --- |
| CGCGGGG | 405 | 0.0 | 36.842415 | 1 |
| CTCGAAC | 8875 | 0.0 | 27.060272 | 1 |
| CGAACAT | 9060 | 0.0 | 23.84126 | 3 |
| TCGAACA | 9670 | 0.0 | 22.484268 | 2 |
| CGGGGCG | 640 | 0.0 | 22.204134 | 1 |
| CGCGGTT | 1415 | 0.0 | 21.592148 | 1 |
| CGCCTCT | 3960 | 0.0 | 21.531282 | 1 |
| CGCGGGT | 860 | 0.0 | 21.481209 | 1 |
| CGCGCAT | 705 | 0.0 | 21.164793 | 1 |
| CGCGTAT | 760 | 0.0 | 19.63313 | 1 |
| AGATCGG | 9305 | 0.0 | 17.896671 | 140-144 |
| CGCGCGG | 245 | 0.0013942157 | 17.400791 | 1 |
| CGGGGAT | 2875 | 0.0 | 15.322783 | 1 |
| CGGGGTT | 2655 | 0.0 | 15.254366 | 1 |
| GCGGGGT | 990 | 0.0 | 15.071898 | 1 |
| CGCCTCG | 1255 | 0.0 | 14.720192 | 1 |
| CGCGTCT | 735 | 5.0931703E-10 | 14.500659 | 1 |
| CCCATAC | 11695 | 0.0 | 14.155603 | 8 |
| GGTTAAC | 15785 | 0.0 | 14.044098 | 1 |
| GCGGGTT | 1515 | 0.0 | 13.600948 | 1 |

Produced by FastQC (version 0.11.5)
