# Supplementary material for: Pitfall Flower Development and Organ Identity of Ceropegia sandersonii (Apocynaceae-Asclepiadoideae)
Source: Plants (Basel). 2020 Dec 14;9(12):1767. doi: 10.3390/plants9121767 (PMC7764971; doi:10.3390/plants9121767)
Supplement: Supplementary file 1 [file plants-09-01767-s001.zip › plants-1003419_Revision1_Supplementary Material/Folder S1_FastQC.zip!/FastQC/CerC38_reverse_paired_fastqc.html]

CerC38\_reverse\_paired.fq FastQC Report 

FastQC Report

Wed 13 Feb 2019  
CerC38\_reverse\_paired.fq

## Summary

- Basic Statistics
- Per base sequence quality
- Per tile sequence quality
- Per sequence quality scores
- Per base sequence content
- Per sequence GC content
- Per base N content
- Sequence Length Distribution
- Sequence Duplication Levels
- Overrepresented sequences
- Adapter Content
- Kmer Content

## Basic Statistics

| Measure | Value |
| --- | --- |
| Filename | CerC38\_reverse\_paired.fq |
| File type | Conventional base calls |
| Encoding | Sanger / Illumina 1.9 |
| Total Sequences | 17567580 |
| Sequences flagged as poor quality | 0 |
| Sequence length | 50-150 |
| %GC | 40 |

## Per base sequence quality

## Per tile sequence quality

## Per sequence quality scores

## Per base sequence content

## Per sequence GC content

## Per base N content

## Sequence Length Distribution

## Sequence Duplication Levels

## Overrepresented sequences

| Sequence | Count | Percentage | Possible Source |
| --- | --- | --- | --- |
| CTGAACTTGTAACTTACTTAAAGAGTGCGAACACCCAACATATTATGCAT | 18225 | 0.10374223427472651 | No Hit |
| CAGAAACTGGGGAAACCAAAAGAAAAAGACGTGGATGCAGGAAGTTCCGG | 17765 | 0.10112377458932875 | No Hit |

## Adapter Content

## Kmer Content

| Sequence | Count | PValue | Obs/Exp Max | Max Obs/Exp Position |
| --- | --- | --- | --- | --- |
| CGCGGGG | 335 | 0.0 | 33.338985 | 1 |
| CTCGAAC | 9340 | 0.0 | 28.698658 | 1 |
| CGCCTCT | 4025 | 0.0 | 27.054277 | 1 |
| CGCGGTT | 1635 | 0.0 | 26.896765 | 1 |
| CGAACAT | 9600 | 0.0 | 25.080278 | 3 |
| TCGAACA | 10150 | 0.0 | 22.969824 | 2 |
| CGGGGGG | 1060 | 0.0 | 21.73128 | 1 |
| CGCGCAT | 900 | 0.0 | 21.716648 | 1 |
| CGCGTAT | 790 | 0.0 | 19.438951 | 1 |
| AGATCGG | 9265 | 0.0 | 18.89906 | 140-144 |
| CGGGCGT | 705 | 0.0 | 18.812292 | 1 |
| GCGGGGG | 1250 | 0.0 | 18.428125 | 1 |
| CGCGCCT | 815 | 0.0 | 17.986177 | 1 |
| CGGGCCC | 590 | 2.3646862E-11 | 17.746655 | 1 |
| CGGGGAT | 2925 | 0.0 | 17.421047 | 1 |
| GATACGA | 10580 | 0.0 | 17.01849 | 7 |
| CGGGCTT | 2465 | 0.0 | 16.707533 | 1 |
| CGGGGCG | 670 | 9.094947E-12 | 16.669493 | 1 |
| CGCCCCT | 1645 | 0.0 | 16.54916 | 1 |
| CCCGCAT | 1310 | 0.0 | 16.518387 | 1 |

Produced by FastQC (version 0.11.5)
