# Supplementary material for: Pitfall Flower Development and Organ Identity of Ceropegia sandersonii (Apocynaceae-Asclepiadoideae)
Source: Plants (Basel). 2020 Dec 14;9(12):1767. doi: 10.3390/plants9121767 (PMC7764971; doi:10.3390/plants9121767)
Supplement: Supplementary file 1 [file plants-09-01767-s001.zip › plants-1003419_Revision1_Supplementary Material/Folder S1_FastQC.zip!/FastQC/CerD11_forward_paired_fastqc.html]

CerD11\_forward\_paired.fq FastQC Report 

FastQC Report

Wed 13 Feb 2019  
CerD11\_forward\_paired.fq

## Summary

- Basic Statistics
- Per base sequence quality
- Per tile sequence quality
- Per sequence quality scores
- Per base sequence content
- Per sequence GC content
- Per base N content
- Sequence Length Distribution
- Sequence Duplication Levels
- Overrepresented sequences
- Adapter Content
- Kmer Content

## Basic Statistics

| Measure | Value |
| --- | --- |
| Filename | CerD11\_forward\_paired.fq |
| File type | Conventional base calls |
| Encoding | Sanger / Illumina 1.9 |
| Total Sequences | 25168657 |
| Sequences flagged as poor quality | 0 |
| Sequence length | 50-150 |
| %GC | 40 |

## Per base sequence quality

## Per tile sequence quality

## Per sequence quality scores

## Per base sequence content

## Per sequence GC content

## Per base N content

## Sequence Length Distribution

## Sequence Duplication Levels

## Overrepresented sequences

No overrepresented sequences

## Adapter Content

## Kmer Content

| Sequence | Count | PValue | Obs/Exp Max | Max Obs/Exp Position |
| --- | --- | --- | --- | --- |
| CGCGGGG | 450 | 7.2759576E-12 | 22.09572 | 1 |
| CGCCTCT | 6220 | 0.0 | 19.18278 | 1 |
| CTCGAAC | 12510 | 0.0 | 14.703986 | 1 |
| AGTCGCT | 9875 | 0.0 | 14.384194 | 6 |
| CGCGTAT | 1210 | 0.0 | 14.087001 | 1 |
| CGCCCCT | 2950 | 0.0 | 13.963639 | 1 |
| CCCGCAT | 4255 | 0.0 | 13.853872 | 1 |
| CGCGCAT | 1120 | 0.0 | 13.316618 | 1 |
| GGTTAAC | 13190 | 0.0 | 12.922874 | 1 |
| GTCGCTT | 11270 | 0.0 | 12.855794 | 7 |
| CGCGCCC | 910 | 9.313226E-10 | 12.487377 | 1 |
| AATCCGC | 5480 | 0.0 | 12.052997 | 5 |
| CGAACAT | 13895 | 0.0 | 11.756064 | 3 |
| TCGAACA | 14685 | 0.0 | 11.413813 | 2 |
| CCCGGCT | 2345 | 0.0 | 11.206023 | 1 |
| CCGCATG | 5040 | 0.0 | 11.132411 | 2 |
| TCGGCTT | 14430 | 0.0 | 11.07411 | 2 |
| AGATCGG | 10360 | 0.0 | 10.82731 | 140-144 |
| CGGGTAT | 3225 | 0.0 | 10.790935 | 1 |
| CGCATGC | 4930 | 0.0 | 10.660497 | 3 |

Produced by FastQC (version 0.11.5)
