# Supplementary material for: Pitfall Flower Development and Organ Identity of Ceropegia sandersonii (Apocynaceae-Asclepiadoideae)
Source: Plants (Basel). 2020 Dec 14;9(12):1767. doi: 10.3390/plants9121767 (PMC7764971; doi:10.3390/plants9121767)
Supplement: Supplementary file 1 [file plants-09-01767-s001.zip › plants-1003419_Revision1_Supplementary Material/Folder S1_FastQC.zip!/FastQC/CerD11_reverse_paired_fastqc.html]

CerD11\_reverse\_paired.fq FastQC Report 

FastQC Report

Wed 13 Feb 2019  
CerD11\_reverse\_paired.fq

## Summary

- Basic Statistics
- Per base sequence quality
- Per tile sequence quality
- Per sequence quality scores
- Per base sequence content
- Per sequence GC content
- Per base N content
- Sequence Length Distribution
- Sequence Duplication Levels
- Overrepresented sequences
- Adapter Content
- Kmer Content

## Basic Statistics

| Measure | Value |
| --- | --- |
| Filename | CerD11\_reverse\_paired.fq |
| File type | Conventional base calls |
| Encoding | Sanger / Illumina 1.9 |
| Total Sequences | 25168657 |
| Sequences flagged as poor quality | 0 |
| Sequence length | 50-150 |
| %GC | 40 |

## Per base sequence quality

## Per tile sequence quality

## Per sequence quality scores

## Per base sequence content

## Per sequence GC content

## Per base N content

## Sequence Length Distribution

## Sequence Duplication Levels

## Overrepresented sequences

No overrepresented sequences

## Adapter Content

## Kmer Content

| Sequence | Count | PValue | Obs/Exp Max | Max Obs/Exp Position |
| --- | --- | --- | --- | --- |
| CGCGGGG | 560 | 0.0 | 32.363106 | 1 |
| CTCGAAC | 12505 | 0.0 | 17.447191 | 1 |
| CGCCTCT | 6410 | 0.0 | 16.311657 | 1 |
| CGCGTTT | 2090 | 0.0 | 16.008839 | 1 |
| CGGGGAT | 5730 | 0.0 | 14.232988 | 1 |
| CGCGCAT | 900 | 5.456968E-12 | 13.94103 | 1 |
| CGAACAT | 13775 | 0.0 | 13.659376 | 3 |
| TCGAACA | 14050 | 0.0 | 13.544133 | 2 |
| CCCGCAT | 4635 | 0.0 | 13.534981 | 1 |
| AGTCGCT | 9075 | 0.0 | 13.361742 | 6 |
| CCCGTCT | 3785 | 0.0 | 12.89131 | 1 |
| CGGGGGG | 1720 | 0.0 | 12.563138 | 1 |
| CTCGAAT | 20345 | 0.0 | 12.162855 | 1 |
| GGTTAAC | 13510 | 0.0 | 12.124879 | 1 |
| AATCCGC | 5405 | 0.0 | 11.990741 | 5 |
| CGGGAAT | 9360 | 0.0 | 11.8409395 | 1 |
| AGATCGG | 10230 | 0.0 | 11.808895 | 140-144 |
| CGCCCCA | 3010 | 0.0 | 11.578929 | 1 |
| CGCGTAT | 1230 | 9.094947E-12 | 11.334171 | 1 |
| GTCGCTT | 11070 | 0.0 | 11.331424 | 7 |

Produced by FastQC (version 0.11.5)
