# Supplementary material for: Pitfall Flower Development and Organ Identity of Ceropegia sandersonii (Apocynaceae-Asclepiadoideae)
Source: Plants (Basel). 2020 Dec 14;9(12):1767. doi: 10.3390/plants9121767 (PMC7764971; doi:10.3390/plants9121767)
Supplement: Supplementary file 1 [file plants-09-01767-s001.zip › plants-1003419_Revision1_Supplementary Material/Folder S1_FastQC.zip!/FastQC/CerD22_forward_paired_fastqc.html]

CerD22\_forward\_paired.fq FastQC Report 

FastQC Report

Wed 13 Feb 2019  
CerD22\_forward\_paired.fq

## Summary

- Basic Statistics
- Per base sequence quality
- Per tile sequence quality
- Per sequence quality scores
- Per base sequence content
- Per sequence GC content
- Per base N content
- Sequence Length Distribution
- Sequence Duplication Levels
- Overrepresented sequences
- Adapter Content
- Kmer Content

## Basic Statistics

| Measure | Value |
| --- | --- |
| Filename | CerD22\_forward\_paired.fq |
| File type | Conventional base calls |
| Encoding | Sanger / Illumina 1.9 |
| Total Sequences | 23684235 |
| Sequences flagged as poor quality | 0 |
| Sequence length | 50-150 |
| %GC | 41 |

## Per base sequence quality

## Per tile sequence quality

## Per sequence quality scores

## Per base sequence content

## Per sequence GC content

## Per base N content

## Sequence Length Distribution

## Sequence Duplication Levels

## Overrepresented sequences

No overrepresented sequences

## Adapter Content

## Kmer Content

| Sequence | Count | PValue | Obs/Exp Max | Max Obs/Exp Position |
| --- | --- | --- | --- | --- |
| CGCGGGG | 560 | 0.0 | 20.288183 | 1 |
| CGCGTTT | 1995 | 0.0 | 13.169523 | 1 |
| AGATCGG | 11920 | 0.0 | 12.319368 | 140-144 |
| CGGGGAT | 5600 | 0.0 | 12.17291 | 1 |
| CCCGCAT | 4045 | 0.0 | 11.586082 | 1 |
| CGCCTCT | 6440 | 0.0 | 11.577496 | 1 |
| CGGGCAT | 8950 | 0.0 | 11.504193 | 1 |
| CGCCTAT | 2390 | 0.0 | 11.290078 | 1 |
| CGGGCGT | 1145 | 2.564775E-10 | 11.162931 | 1 |
| CGCGTAT | 1145 | 2.564775E-10 | 11.162931 | 1 |
| CGGGGGT | 1760 | 0.0 | 10.489913 | 1 |
| CGCCCAT | 6890 | 0.0 | 10.409104 | 1 |
| CGGGGAC | 4955 | 0.0 | 10.318108 | 1 |
| CTCGAAC | 12665 | 0.0 | 10.148096 | 1 |
| CGCGTGT | 1050 | 1.02065314E-7 | 10.144092 | 1 |
| CCCGCCT | 3945 | 0.0 | 10.079807 | 1 |
| CGCCCCT | 3130 | 0.0 | 9.982045 | 1 |
| CCCGCTT | 3285 | 0.0 | 9.943372 | 1 |
| CGGGAAT | 10665 | 0.0 | 9.853989 | 1 |
| CGGGCCT | 2550 | 0.0 | 9.7462845 | 1 |

Produced by FastQC (version 0.11.5)
