# Supplementary material for: Pitfall Flower Development and Organ Identity of Ceropegia sandersonii (Apocynaceae-Asclepiadoideae)
Source: Plants (Basel). 2020 Dec 14;9(12):1767. doi: 10.3390/plants9121767 (PMC7764971; doi:10.3390/plants9121767)
Supplement: Supplementary file 1 [file plants-09-01767-s001.zip › plants-1003419_Revision1_Supplementary Material/Folder S1_FastQC.zip!/FastQC/CerD22_reverse_paired_fastqc.html]

CerD22\_reverse\_paired.fq FastQC Report 

FastQC Report

Wed 13 Feb 2019  
CerD22\_reverse\_paired.fq

## Summary

- Basic Statistics
- Per base sequence quality
- Per tile sequence quality
- Per sequence quality scores
- Per base sequence content
- Per sequence GC content
- Per base N content
- Sequence Length Distribution
- Sequence Duplication Levels
- Overrepresented sequences
- Adapter Content
- Kmer Content

## Basic Statistics

| Measure | Value |
| --- | --- |
| Filename | CerD22\_reverse\_paired.fq |
| File type | Conventional base calls |
| Encoding | Sanger / Illumina 1.9 |
| Total Sequences | 23684235 |
| Sequences flagged as poor quality | 0 |
| Sequence length | 50-150 |
| %GC | 41 |

## Per base sequence quality

## Per tile sequence quality

## Per sequence quality scores

## Per base sequence content

## Per sequence GC content

## Per base N content

## Sequence Length Distribution

## Sequence Duplication Levels

## Overrepresented sequences

No overrepresented sequences

## Adapter Content

## Kmer Content

| Sequence | Count | PValue | Obs/Exp Max | Max Obs/Exp Position |
| --- | --- | --- | --- | --- |
| CGCGGGG | 605 | 0.0 | 26.514502 | 1 |
| CCCGCAT | 4050 | 0.0 | 14.121143 | 1 |
| AGATCGG | 11765 | 0.0 | 12.963203 | 140-144 |
| CGCGTTT | 1700 | 0.0 | 12.718145 | 1 |
| CGCCTCT | 6495 | 0.0 | 12.5637045 | 1 |
| CGCGTGT | 1115 | 1.8189894E-12 | 12.510255 | 1 |
| CGGGGAT | 5550 | 0.0 | 12.189609 | 1 |
| CGGGCCC | 1120 | 1.8189894E-11 | 11.831685 | 1 |
| CCCGTCT | 4610 | 0.0 | 11.800616 | 1 |
| CGCGTAT | 1200 | 5.456968E-12 | 11.624111 | 1 |
| CGGGCAT | 8390 | 0.0 | 11.47171 | 1 |
| CGGGGGG | 1775 | 0.0 | 11.394903 | 1 |
| GCCGGGT | 2475 | 0.0 | 11.271866 | 1 |
| CGGGACT | 4455 | 0.0 | 11.271866 | 1 |
| CGCCCTT | 8020 | 0.0 | 11.218282 | 1 |
| CGGGGCG | 1010 | 6.373739E-9 | 11.04866 | 1 |
| CGCGCAT | 1095 | 1.9481377E-9 | 10.82794 | 1 |
| CGCCCCT | 3360 | 0.0 | 10.7938175 | 1 |
| CGGGAAT | 10865 | 0.0 | 10.7842655 | 1 |
| CGGGCGT | 1110 | 2.4392648E-9 | 10.681616 | 1 |

Produced by FastQC (version 0.11.5)
