# Supplementary material for: Pitfall Flower Development and Organ Identity of Ceropegia sandersonii (Apocynaceae-Asclepiadoideae)
Source: Plants (Basel). 2020 Dec 14;9(12):1767. doi: 10.3390/plants9121767 (PMC7764971; doi:10.3390/plants9121767)
Supplement: Supplementary file 1 [file plants-09-01767-s001.zip › plants-1003419_Revision1_Supplementary Material/Folder S1_FastQC.zip!/FastQC/CerD33_forward_paired_fastqc.html]

CerD33\_forward\_paired.fq FastQC Report 

FastQC Report

Wed 13 Feb 2019  
CerD33\_forward\_paired.fq

## Summary

- Basic Statistics
- Per base sequence quality
- Per tile sequence quality
- Per sequence quality scores
- Per base sequence content
- Per sequence GC content
- Per base N content
- Sequence Length Distribution
- Sequence Duplication Levels
- Overrepresented sequences
- Adapter Content
- Kmer Content

## Basic Statistics

| Measure | Value |
| --- | --- |
| Filename | CerD33\_forward\_paired.fq |
| File type | Conventional base calls |
| Encoding | Sanger / Illumina 1.9 |
| Total Sequences | 22236452 |
| Sequences flagged as poor quality | 0 |
| Sequence length | 50-150 |
| %GC | 41 |

## Per base sequence quality

## Per tile sequence quality

## Per sequence quality scores

## Per base sequence content

## Per sequence GC content

## Per base N content

## Sequence Length Distribution

## Sequence Duplication Levels

## Overrepresented sequences

No overrepresented sequences

## Adapter Content

## Kmer Content

| Sequence | Count | PValue | Obs/Exp Max | Max Obs/Exp Position |
| --- | --- | --- | --- | --- |
| CGCGGGG | 525 | 3.6379788E-12 | 20.277489 | 1 |
| CCCGCAT | 3990 | 0.0 | 16.720036 | 1 |
| CGCCTCT | 6080 | 0.0 | 13.073644 | 1 |
| CGCGCAT | 1115 | 1.8189894E-12 | 12.730262 | 1 |
| CGGGCAT | 8065 | 0.0 | 12.495862 | 1 |
| CTCGAAC | 12755 | 0.0 | 12.074286 | 1 |
| CGCCCAT | 6160 | 0.0 | 11.751727 | 1 |
| AATCCGC | 5895 | 0.0 | 11.557652 | 5 |
| CGGGGAT | 5060 | 0.0 | 11.361004 | 1 |
| AGATCGG | 10265 | 0.0 | 11.233273 | 140-144 |
| CGGGAAT | 9730 | 0.0 | 11.014031 | 1 |
| CGCGCAC | 720 | 1.5977628E-5 | 10.842824 | 1 |
| CGCATGC | 4650 | 0.0 | 10.836465 | 3 |
| CGGGCGT | 1185 | 4.6929927E-10 | 10.780437 | 1 |
| CGCGTTT | 1975 | 0.0 | 10.7804365 | 1 |
| CGCCCCT | 2900 | 0.0 | 10.768045 | 1 |
| CCGCATG | 4690 | 0.0 | 10.744043 | 2 |
| CGAACAT | 12700 | 0.0 | 10.673624 | 3 |
| CGCACAT | 3880 | 0.0 | 10.426183 | 1 |
| CGCCCTT | 7880 | 0.0 | 10.177343 | 1 |

Produced by FastQC (version 0.11.5)
