# Supplementary material for: Pitfall Flower Development and Organ Identity of Ceropegia sandersonii (Apocynaceae-Asclepiadoideae)
Source: Plants (Basel). 2020 Dec 14;9(12):1767. doi: 10.3390/plants9121767 (PMC7764971; doi:10.3390/plants9121767)
Supplement: Supplementary file 1 [file plants-09-01767-s001.zip › plants-1003419_Revision1_Supplementary Material/Folder S1_FastQC.zip!/FastQC/CerD33_reverse_paired_fastqc.html]

CerD33\_reverse\_paired.fq FastQC Report 

FastQC Report

Wed 13 Feb 2019  
CerD33\_reverse\_paired.fq

## Summary

- Basic Statistics
- Per base sequence quality
- Per tile sequence quality
- Per sequence quality scores
- Per base sequence content
- Per sequence GC content
- Per base N content
- Sequence Length Distribution
- Sequence Duplication Levels
- Overrepresented sequences
- Adapter Content
- Kmer Content

## Basic Statistics

| Measure | Value |
| --- | --- |
| Filename | CerD33\_reverse\_paired.fq |
| File type | Conventional base calls |
| Encoding | Sanger / Illumina 1.9 |
| Total Sequences | 22236452 |
| Sequences flagged as poor quality | 0 |
| Sequence length | 50-150 |
| %GC | 41 |

## Per base sequence quality

## Per tile sequence quality

## Per sequence quality scores

## Per base sequence content

## Per sequence GC content

## Per base N content

## Sequence Length Distribution

## Sequence Duplication Levels

## Overrepresented sequences

No overrepresented sequences

## Adapter Content

## Kmer Content

| Sequence | Count | PValue | Obs/Exp Max | Max Obs/Exp Position |
| --- | --- | --- | --- | --- |
| CGCGGGG | 525 | 0.0 | 35.92725 | 1 |
| CCCGCAT | 3790 | 0.0 | 17.879362 | 1 |
| CGGGCCC | 1025 | 0.0 | 14.994027 | 1 |
| CGCCTCT | 6805 | 0.0 | 13.961441 | 1 |
| CGGGGAT | 5615 | 0.0 | 13.6855545 | 1 |
| CGCGTTT | 1950 | 0.0 | 13.613459 | 1 |
| CGGGCAT | 7990 | 0.0 | 13.464599 | 1 |
| CTCGAAC | 12950 | 0.0 | 13.21648 | 1 |
| CGCGTAT | 1080 | 9.094947E-12 | 12.2899275 | 1 |
| CGGGGGG | 1710 | 0.0 | 12.255883 | 1 |
| CGCCCCT | 3200 | 0.0 | 12.2252445 | 1 |
| AGATCGG | 10125 | 0.0 | 11.964291 | 140-144 |
| CGCGCAT | 1115 | 1.6370905E-11 | 11.904145 | 1 |
| CCGCATG | 4195 | 0.0 | 11.823494 | 2 |
| AATCCGC | 5940 | 0.0 | 11.640393 | 5 |
| CGCATGC | 4240 | 0.0 | 11.530578 | 3 |
| CGCGTGT | 1045 | 8.749339E-10 | 11.364546 | 1 |
| CGGGGCT | 3565 | 0.0 | 11.169527 | 1 |
| CGCCCTT | 7200 | 0.0 | 10.96391 | 1 |
| CGCCCAT | 6075 | 0.0 | 10.924381 | 1 |

Produced by FastQC (version 0.11.5)
